# Molecular mechanisms of antibiotic resistance in Gram-positive and Gram-negative bacteria - a narrative review

**DOI:** 10.3389/fcimb.2026.1877136

**Published:** 2026-08-10

**Authors:** Denisa Claudia Tonco, Cristina Gavrilovici, Ancuta Lupu, Leonard Iosif Pertea, Ileana Ioniuc, Alice Grudnicki, Sorana Caterina Anton, Alin Horatiu Nedelcu, Tania Rusu, Costica Mitrofan, Manuel Florin Rosu, Ionela Daniela Morariu, Emil Anton, Elena Cristina Mitrofan, Vasile Valeriu Lupu

**Affiliations:** 1 Grigore T. Popa University of Medicine and Pharmacy, Iasi, Romania; 2 Regina Maria Healthcare Campus, Iasi, Romania; 3 Clinic of Pulmonary Disease, Iasi, Romania

**Keywords:** antibiotic resistance, biofilm, efflux pumps, ESKAPE pathogens, Gram-negative pathogens, Gram-positive pathogens, lariocidin

## Abstract

Antimicrobial resistance (AMR) represents one of the most pressing global public health challenges of the 21st century, driven by the widespread inappropriate use of antibiotics and the remarkable adaptive capacity of pathogenic bacteria This narrative review provides an integrated comparative analysis of AMR mechanisms across Gram-positive and Gram-negative bacteria, with particular emphasis on clinically significant ESKAPE pathogens. The four principal resistance mechanisms: enzymatic drug inactivation, reduced drug uptake, target site modification, and active efflux, are examined comparatively across both organism groups, with emphasis on how their molecular basis and clinical significance differ between them. Biofilm formation is further addressed as a resistance-amplifying strategy, with discussion of organism-specific differences in matrix composition between Gram-positive and Gram-negative pathogens. Current and emerging diagnostic approaches for AMR detection are reviewed in relation to their differential applicability across organism groups, followed by a discussion of pharmacokinetic/pharmacodynamic optimization as a resistance-prevention strategy. Finally, lariocidin, a structurally novel ribosome-targeting lasso peptide with broad-spectrum activity against both Gram-positive and Gram-negative multidrug-resistant pathogens, is presented as an emerging therapeutic scaffold that circumvents resistance mechanisms operating across both bacterial groups. Where relevant, illustrative examples from pediatric populations are discussed, highlighting how diagnostic challenges and empirical prescribing may compound the impact of resistance in this group. Addressing AMR requires coordinated global efforts that integrate surveillance, research, policy-making, and education to ensure the continued efficacy of antimicrobial treatments.

## 1 Introduction

Antimicrobial resistance (AMR) is defined as the ability of bacteria, viruses, fungi, and parasites to resist the effects of antimicrobial agents to which they were previously susceptible (Yu et al., 2023). Although antibiotic-resistant bacteria have existed for millions of years and were historically confined largely to environmental settings, the extensive use of antibiotics over the past seven decades in agriculture, livestock production, veterinary care and human medicine has created substantial selective pressure favoring resistant strains (Medernach and Logan, 2018). Consequently, standard treatments, including antibiotics and other antimicrobials, become ineffective, leading to persistent infections that are more difficult or, in some cases, impossible to treat. This significantly increases the risk of disease transmission, severe clinical outcomes, and mortality. AMR represents a critical global public health challenge, across all age groups, including children. In the EU/EEA, more than 35,000 deaths were estimated to be attributable to infections caused by antibiotic-resistant bacteria in 2020 (European Centre for Disease Prevention and Control, 2024).

Due to their immature immune systems, children are particularly vulnerable to infectious diseases such as pneumonia and meningitis, which are commonly managed with antibiotic therapy (Medernach and Logan, 2018; Yu et al., 2023). However, limited understanding of resistance mechanisms in prevalent pediatric pathogens, along with a scarcity of pediatric-specific data, has contributed to the overuse and misuse of antibiotics thereby exacerbating the threat of AMR in pediatric infections as a significant public health concern (Duceac et al., 2018; Yu et al., 2023). Similar concerns have been reported in pediatric *Helicobacter pylori* infections, where increasing clarithromycin resistance has been associated with reduced eradication success rates and growing therapeutic challenges (Rosu et al., 2022). Historically, Gram-negative bacteria were more typically associated with life-threatening infections than Gram-positive bacteria (Duceac et al., 2018; Sharmila Devi et al., 2024).

AMR is directly associated with substantial pathogen-specific mortality. According to the Global Burden of Bacterial Antimicrobial Resistance study, an estimated 4.95 million deaths were associated with bacterial AMR in 2019, including 1.27 million deaths directly attributable to resistant infections, with the highest burden linked to Escherichia coli, Staphylococcus aureus, Klebsiella pneumoniae, Streptococcus pneumoniae, Acinetobacter baumannii, and Pseudomonas aeruginosa (Murray et al., 2022). Recent global analyses have further quantified the epidemiological impact of AMR beyond earlier estimates. A landmark *Lancet* analysis of AMR trends from 1990 to 2021 found that at least one million deaths occurred annually due to resistant infections, with models projecting that annual AMR deaths may reach up to ~1.91 million by 2050 and that more than ~39 million deaths could occur globally between 2025 and 2050 if current trends persist (GBD 2021 Antimicrobial Resistance Collaborators, 2024).

The growing prevalence of AMR has led to increased reliance on second-line and last-resort therapies, which are often more expensive, less well tolerated, and sometimes less effective. This shift significantly amplifies the economic burden on healthcare systems. In Europe alone, AMR is estimated to generate more than nine billion euros in healthcare-related costs annually. In the United States, resistant infections account for approximately 20 billion dollars in excess direct healthcare expenditures each year, in addition to an estimated 35 billion dollars in productivity losses (Dadgostar, 2019).

The global initiative to combat AMR has intensified significantly, focusing both on the development of novel antimicrobial agents and the modification of existing compounds to address the rise of resistant pathogens. AMR emerges when bacteria evade the effects of antibiotics through various mechanisms, such as enzymatic inactivation of the drug, active efflux from the cell, or structural modifications that prevent antibiotic binding (Sharmila Devi et al., 2024). Bacterial resistance can be broadly understood as either intrinsic or acquired. Intrinsic resistance reflects inherent structural or physiological traits that naturally limit antibiotic activity. Acquired resistance, in contrast, develops either through spontaneous chromosomal mutations that modify antibiotic targets or alter protein expression, or through the acquisition of exogenous resistance determinants via horizontal gene transfer. The latter is mediated by mobile genetic elements such as plasmids and transposons and occurs through transformation, transduction, and most importantly, conjugation, which plays a central role in the rapid spread of resistance across species and environment (Domingues et al., 2012; Munita and Arias, 2016; Breijyeh et al., 2020).

In response to the growing threat of antimicrobial resistance, the World Health Organization launched the Global Action Plan on AMR in 2015 (WHO, 2015). Subsequently, the World Health Organization published a global priority list of antibiotic-resistant pathogens, classifying them as critical, high, or medium priority based on the urgency of developing new antibiotics. This prioritization was established using a multi-criteria decision analysis that considered mortality, disease burden, resistance trends, transmissibility, preventability, treatability, and the status of the antibacterial development pipeline, informed by expert panel input. The list serves to guide research priorities and focus resources on pathogens that pose the greatest threat to global health (Rajput et al., 2024; Sati et al., 2025).

This narrative review was conducted based on a comprehensive literature search using key terms such as antimicrobial resistance, antibiotic resistance, multidrug-resistant bacteria, resistance mechanisms, biofilm, beta-lactamases, efflux pumps, and ESKAPE pathogens. The literature search was performed across major scientific databases, including PubMed, MEDLINE, Scopus, Web of Science and Google Scholar, to ensure broad coverage of biomedical and microbiological research. Additional relevant articles were identified through manual screening of reference lists from selected publications. The scope of this review is to provide an overview of the major molecular and cellular mechanisms underlying bacterial antibiotic resistance, with particular emphasis on multidrug-resistant pathogens of clinical importance, and to discuss their implications for antimicrobial therapy, infection control, and future drug development.

While numerous reviews have addressed AMR, many focus either on specific bacterial groups, isolated resistance mechanisms, or particular clinical settings. In contrast, the present narrative review provides an integrated and comparative analysis of molecular resistance mechanisms across both Gram-positive and Gram-negative pathogens, with particular emphasis on clinically relevant multidrug-resistant ESKAPE organisms. In addition to detailing enzymatic inactivation, target modification, reduced permeability, and efflux-mediated resistance, this review contextualizes these mechanisms within current epidemiological trends and clinical implications, with brief illustrative reference to pediatric infections. Furthermore, we highlight emerging therapeutic perspectives, including novel antimicrobial scaffolds such as ribosome-targeting lasso peptides. The escalating molecular complexity of AMR, particularly among high-priority ESKAPE pathogens, has transformed once-manageable infections into major therapeutic challenges (Murray et al., 2022; GBD 2021 Antimicrobial Resistance Collaborators, 2024). A mechanistic understanding of resistance is therefore not merely descriptive but foundational to the development of rapid diagnostics, stewardship strategies, and structurally novel antimicrobial agents (WHO, 2015; Munita and Arias, 2016). This review integrates molecular mechanisms with clinical implications and emerging therapeutic perspectives to provide a coherent translational framework for addressing antimicrobial resistance.

## 2 Gram-negative versus Gram-positive bacteria - molecular foundations of antimicrobial resistance

The molecular basis of AMR differs substantially between Gram-positive and Gram-negative bacteria, reflecting fundamental differences in cell envelope architecture, genetic plasticity, and ecological niche (Munita and Arias, 2016; Breijyeh et al., 2020; Sharmila Devi et al., 2024). Enzymatic degradation, particularly via β-lactamases, represents one of the most clinically significant mechanisms, with diverse classes capable of hydrolyzing penicillins, cephalosporins, monobactams, and carbapenems (Bush and Jacoby, 2010). Target modification, often driven by chromosomal mutations or horizontally acquired genes, alters the binding affinity of antibiotics to critical structures such as penicillin-binding proteins, ribosomal RNA, DNA gyrase, or dihydrofolate reductase, thereby preserving essential cellular functions while reducing drug susceptibility (Munita and Arias, 2016). In Gram-negative bacteria, intrinsic structural barriers such as the outer membrane, combined with porin alterations, further restrict antibiotic entry, whereas multidrug efflux pumps from families such as RND, MFS, and ABC actively expel diverse antimicrobial agents, contributing to multidrug resistance phenotypes. Importantly, these mechanisms rarely occur in isolation; their coexistence within single strains, frequently facilitated by mobile genetic elements, underlies the emergence of highly resilient multidrug-resistant pathogens that pose substantial therapeutic challenges (Reygaert, 2018).

Gram-negative bacteria are characterized by a complex, multi-layered cell envelope comprising three distinct components arranged from the exterior inward: the outer membrane (OM), the peptidoglycan layer, and the inner (cytoplasmic) membrane (IM). The OM and IM encapsulate the periplasmic space, an aqueous compartment housing the peptidoglycan layer. Each structural element plays a crucial role in maintaining cellular viability and contributes uniquely to the integrity and function of the bacterial envelope (Collet et al., 2020; Gauba and Rahman, 2023). The inner membrane is a conventional phospholipid bilayer, while the outer membrane is compositionally asymmetric; its inner leaflet is composed of phospholipids, whereas its outer leaflet contains lipopolysaccharides. Between these two membranes lies the periplasmic space, a gel-like compartment that comprises approximately 10–20% of the cell’s total volume. Within this compartment resides a thin peptidoglycan layer, also known as the cell wall, which is formed by repeating units of N-acetylglucosamine and N-acetylmuramic acid, interconnected by short peptide bridges (Typas et al., 2012; Collet et al., 2020).

The OM is the first structural barrier encountered when transitioning from the external environment to the interior of a Gram-negative bacterium. This membrane is a defining feature of Gram-negative organisms and is absent in Gram-positive bacteria. Although the OM resembles other biological membranes in its bilayer structure, its composition is notably distinct. Phospholipids are confined to the inner leaflet, while the outer leaflet predominantly consists of glycolipids, particularly lipopolysaccharides (LPS). LPS is a potent immunostimulatory molecule that activates the host’s innate immune system and is the primary mediator of endotoxic shock associated with Gram-negative septicemia (Nikaido, 2003; Sun et al., 2022).

Lipoproteins are embedded in the inner leaflet of the OM, where they are anchored via lipid modifications linked to an N-terminal cysteine residue. Gram-negative bacteria, such as *Escherichia coli*, are capable of producing a wide array of lipoproteins. *E. coli* alone expresses approximately 80 distinct variants. These molecules serve various essential functions, including roles in virulence, peptidoglycan remodeling and synthesis, envelope architecture, stress response, cell division, and OM biogenesis (El Rayes et al., 2021; Sun et al., 2022). Among OM constituents, outer membrane proteins (OMPs) are the most abundant and typically adopt β-barrel conformations. These β-barrels consist of an even number of antiparallel β-strands that span the membrane, forming asymmetric structures with short periplasmic loops and extended extracellular loops. Positioned on the bacterial surface, β-barrel OMPs serve as the initial interface with the environment and perform diverse roles. These include regulating the uptake of hydrophilic antibiotics and nutrients, maintaining membrane stability, mediating adhesion during pathogenesis, and facilitating the transport of molecules such as siderophores, lipases, and proteases (Rollauer et al., 2015; El Rayes et al., 2021).

Gram-negative bacteria increasingly exhibit resistance to antibiotics through diverse mechanisms. While some possess intrinsic resistance, many acquire resistance genes via mobile genetic elements (MGEs) transferred through horizontal gene transfer (HGT). HGT enables rapid adaptation by transferring large DNA segments, including pathogenicity islands, between bacteria via transformation, transduction, or, most commonly, conjugation. This process facilitates the emergence of multidrug-resistant “superbugs” carrying numerous resistance genes on plasmids. AMR mechanisms generally fall into four categories: enzymatic drug inactivation, reduced drug uptake, modification of the drug target, and enhanced drug efflux (Munita and Arias, 2016; Dell’Annunziata et al., 2021; Tao et al., 2022).

On the other hand, Gram-positive bacteria are characterized by their ability to retain crystal violet dye, appearing blue under microscopic examination due to their thick peptidoglycan (PG) layer. Unlike Gram-negative bacteria, Gram-positive bacteria lack an outer membrane, and their robust PG layer envelops the plasma membrane, providing protection against environmental stressors. Other structural differences are listed in **Table 1**. The synthesis, thickness, chemical composition, and degree of cross-linking of peptidoglycan are key determinants of bacterial cell shape and morphology. Gram-positive bacteria may exhibit spherical shapes (e.g., *Staphylococcus* and *Streptococcus* species), rod-like structures (e.g., *Bacillus subtilis*), or form branching filaments. In addition to peptidoglycan, the cell wall incorporates anionic polymers known as teichoic acids, membrane-associated proteins involved in sensing and molecular transport, and capsular polysaccharides that are covalently linked to the PG matrix (Rice, 2006; Yang et al., 2016; Rajagopal and Walker, 2017).

**Table 1:** Key differences between Gram-positive and Gram-negative bacteria (Nikaido, 2003; Rice, 2006; Munita and Arias, 2016; Yang et al., 2016; El Rayes et al., 2021; Sun et al., 2022).

Characteristic	Gram-positive bacteria	Gram-negative bacteria
Cell Wall Structure	Thick peptidoglycan layer	Thin peptidoglycan layer + outer membrane
Teichoic Acids	Present	Absent
Outer Membrane	Absent	Present (contains lipopolysaccharides)
Periplasmic Space	Usually absent or minimal	Present and well-defined
Staining	Retain crystal violet (purple)	Do not retain crystal violet (pink/red after counterstain)
Flagella Structure	2 basal body rings	4 basal body rings
Susceptibility to Lysozyme	High	Low
Examples	*Staphylococcus aureus*, *Streptococcus pneumoniae*	*Escherichia coli*, *Pseudomonas aeruginosa, Klebsiella pneumoniae*

### 2.1 Enzymatic drug inactivation: shared strategies, distinct clinical impact across Gram positive and Gram-negative bacteria

**Figure 1:**
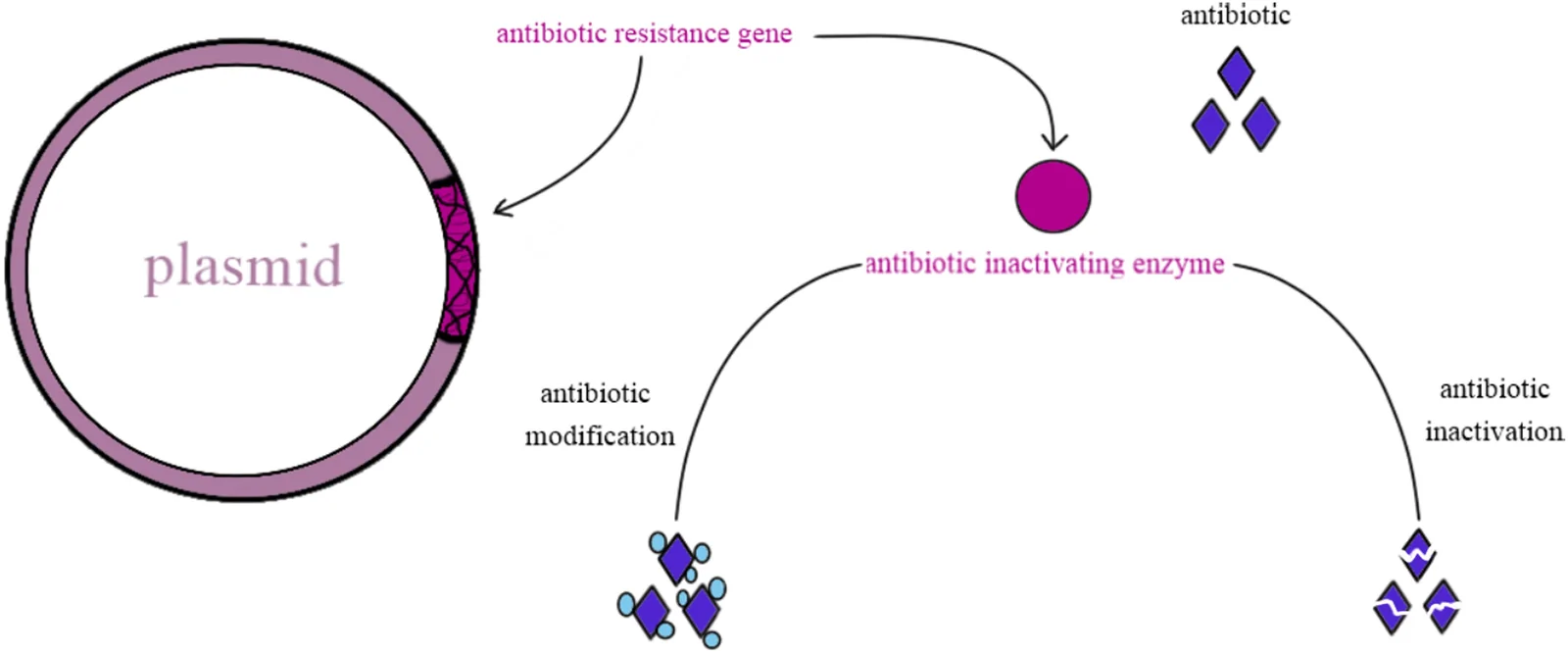
Enzymatic drug inactivation (adapted from 31 32).

Enzymatic drug inactivation is among the most clinically significant resistance mechanisms in both Gram-negative and Gram-positive bacteria, involving either degradation of the antibiotic’s active structure or chemical modification that prevents target binding (De Oliveira et al., 2020). The representation of the mechanism is illustrated in **Figure 1**. In Gram-negative bacteria, these enzymes are especially prevalent and function primarily through two strategies: enzymatic degradation of the antibiotic’s active site (e.g., β-lactamases that hydrolyze the β-lactam ring) or chemical modification of the drug to prevent interaction with its bacterial target (e.g., aminoglycoside-modifying enzymes (AMEs), which alter hydroxyl or amino groups on the molecule) (Egorov et al., 2018; De Oliveira et al., 2020).

β-Lactamase enzymes were first discovered shortly after the isolation of penicillin. To date, more than 2,600 distinct β-lactamases have been identified, each conferring resistance to one or more classes of β-lactam antibiotics, including penicillins, cephalosporins, monobactams, and carbapenems. Localized within the periplasmic space, β-lactamases inactivate these antibiotics by hydrolyzing them before the drugs can interact with their targets, namely, the penicillin-binding proteins (PBPs) located in the bacterial cell wall (Naas et al., 2017; De Oliveira et al., 2020).

β-Lactamases are classified using two principal systems: one based on molecular structure and another on functional activity. Structurally, they are divided into four classes (A–D) according to conserved sequence motifs. Classes A, C, and D are serine β-lactamases that hydrolyze β-lactams via a covalent acyl-enzyme intermediate, whereas class B enzymes are zinc-dependent metallo-β-lactamases (Bonomo, 2017). In the functional classification system, β-lactamases are grouped according to substrate profile and inhibitor susceptibility. Group 1 enzymes (molecular class C) are primarily chromosomal cephalosporinases commonly found in Enterobacterales and are typically resistant to clavulanic acid. Group 2 comprises serine β-lactamases (classes A and D), which hydrolyze penicillins and various other β-lactams with variable inhibitor susceptibility. Group 3 includes metallo-β-lactamases (class B), zinc-dependent enzymes that hydrolyze most β-lactams except monobactams and are not inhibited by clavulanic acid but can be affected by metal chelators such as EDTA (Bush and Jacoby, 2010; Bonomo, 2017; Tooke et al., 2019; Gauba and Rahman, 2023). Carbapenem resistance in Gram-negatives is mainly driven by β-lactamases, especially carbapenemases that hydrolyze the β-lactam ring of carbapenem antibiotics, rendering them inactive. These enzymes include class A (e.g., KPC), class B metallo-β-lactamases (e.g., NDM), and class D enzymes (e.g., OXA-type carbapenemases). Their diverse catalytic mechanisms allow broad hydrolysis of carbapenems and other β-lactams, and are a principal factor in clinical resistance across *Enterobacterales*, *Pseudomonas aeruginosa*, and *Acinetobacter* species — substantially limiting therapeutic options (Mourabiti et al., 2025).

In Gram-positive bacteria, enzymatic inactivation follows the same two strategies. The most common form is enzymatic degradation by β-lactamases, which hydrolyze the β-lactam ring and inactivate β-lactam antibiotics. The second major strategy is drug modification through the enzymatic transfer of chemical groups (acetyl, phosphoryl, or adenyl) to the antibiotic molecule. This is commonly observed in resistance to aminoglycosides, chloramphenicol, and streptogramins. AMEs including acetyltransferases, phosphotransferases, and nucleotidyltransferases, interfere with antibiotic-target binding, thereby impairing drug efficacy and contributing to high levels of resistance in Gram-positive pathogens (Blair et al., 2015; Jubeh et al., 2020; Uddin et al., 2021).

### 2.2 Reduced drug uptake: outer membrane barriers in Gram-negative versus cell wall modifications in Gram-positive bacteria

Many antibiotics act on intracellular or membrane-associated targets and must therefore penetrate bacterial envelope barriers to exert their effects. The relative contribution of this mechanism, and the structural basis underlying it, differ substantially between Gram-negative and Gram-positive organisms (Blair et al., 2015; Munita and Arias, 2016).

**Figure 2:**
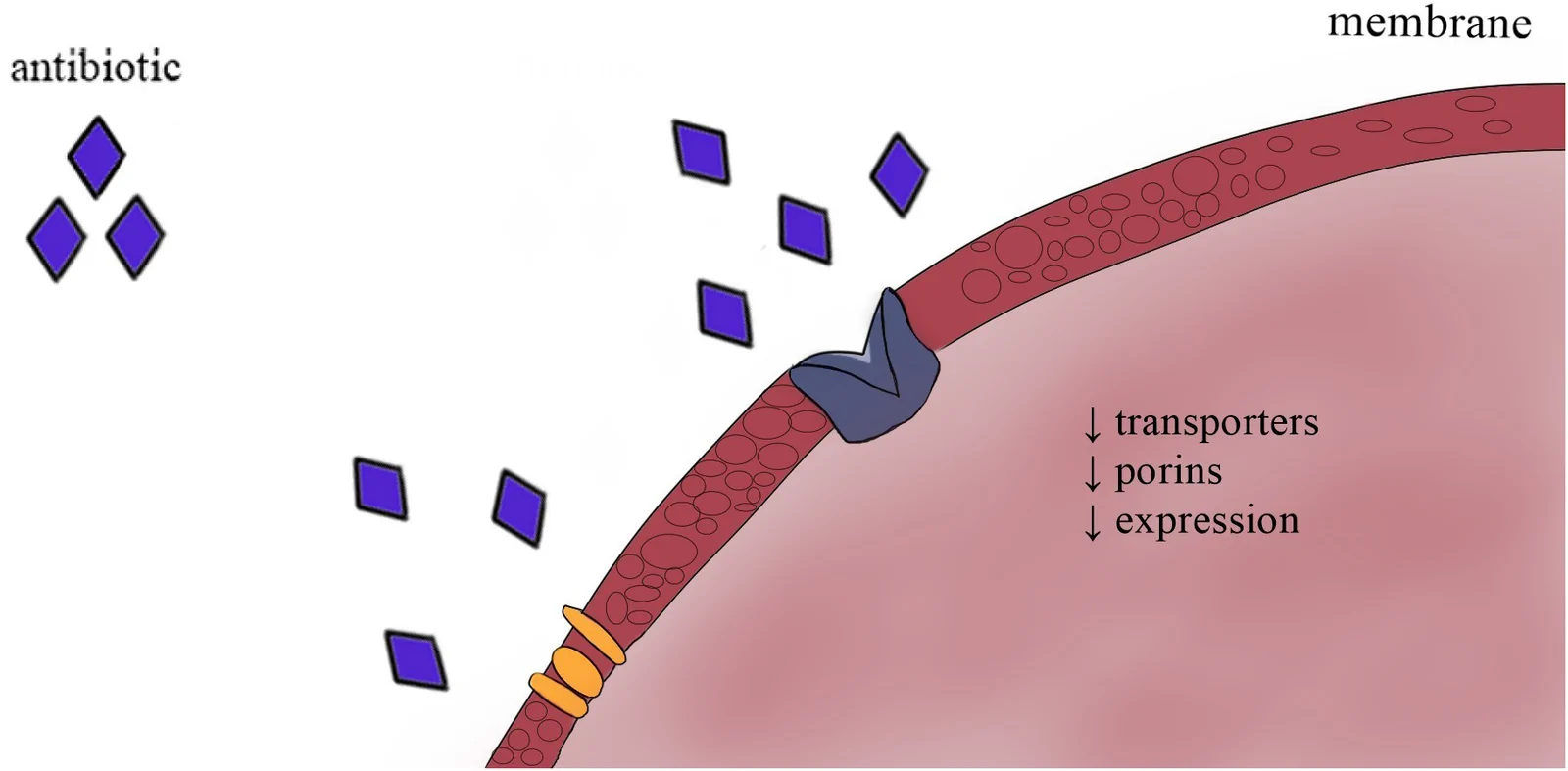
Reduced drug uptake (adapted from 40-42).

In Gram-negative bacteria, the outer membrane constitutes a critical permeability barrier (**Figure 2**), limiting antibiotic entry (Blair et al., 2015; Munita and Arias, 2016). Hydrophilic agents such as β-lactams and certain fluoroquinolones rely on porin channels to cross this barrier, making susceptibility highly dependent on porin expression and function. Alterations in porin type, reduced expression, or structural modifications can diminish drug uptake and contribute to resistance. A well-known example is the intrinsic resistance of Gram-negative organisms to vancomycin, which cannot traverse the outer membrane. Porin-mediated resistance is often associated with low-level decreased susceptibility and may act synergistically with additional mechanisms, including efflux pump overexpression (Munita and Arias, 2016; Elshobary et al., 2025). In *Pseudomonas aeruginosa*, resistance to imipenem is frequently linked to mutations in the oprD gene, which encodes a porin involved in the uptake of basic amino acids and carbapenems (Quinn et al., 1986). These mutations can emerge during therapy and may lead to high-level resistance, especially when combined with efflux pump overexpression or carbapenemase production. Similarly, in *Klebsiella pneumoniae*, post-treatment isolates often exhibit a shift from OmpK35 to OmpK36 porins with narrower channels, resulting in significantly reduced β-lactam susceptibility. Comparable resistance patterns have also been observed in *Enterobacter cloacae*, *Salmonella* spp., *Neisseria gonorrhoeae*, and *Acinetobacter baumannii* (Doménech-Sánchez et al., 2003). Another critical mechanism in Gram-negative resistance involves loss or alteration of outer membrane porins that normally facilitate the entry of carbapenems. Mutations or deletions in porin genes (e.g., *ompK35*, *ompK36* in *Klebsiella pneumoniae*) restrict carbapenem penetration into the periplasmic space, effectively reducing intracellular drug concentrations and contributing to high-level resistance, especially when combined with β-lactamase production (Mourabiti et al., 2025).

### 2.3 Modification of drug target: carbapenem and fluoroquinolone resistance in Gram-negatives versus PBP and ribosomal alterations in Gram-positives

**Figure 3:**
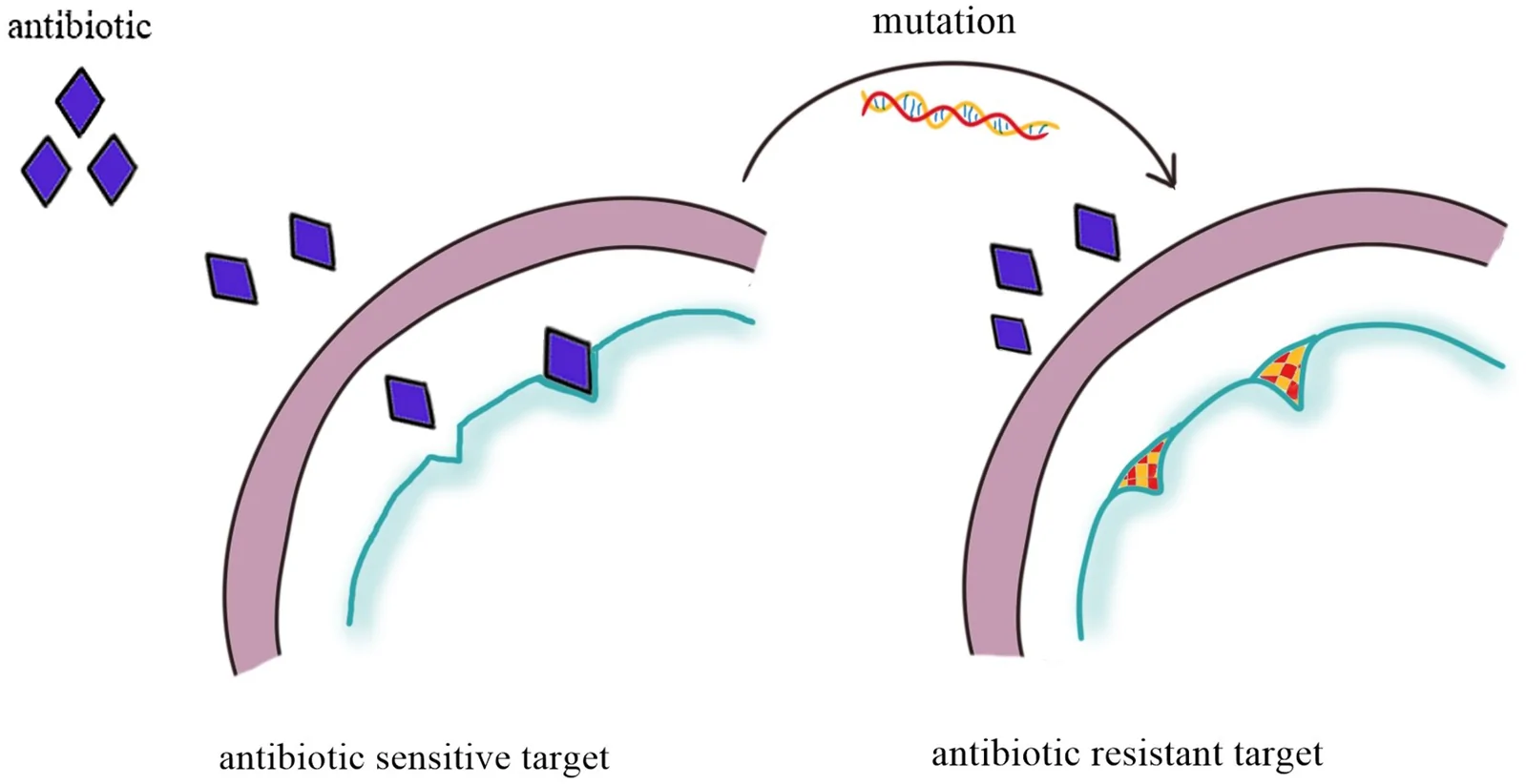
Modification of drug target (adapted from 49, 51).

Modification of the drug target is a key resistance mechanism that reduces the drug’s ability to bind effectively, thereby diminishing its potency. While target site modification occurs in both Gram-positive and Gram-negative bacteria, the specific targets affected and the clinical consequences differ markedly between the two groups (Blair et al., 2015; Munita and Arias, 2016; Reygaert, 2018). However, only mutations that impair antibiotic binding while preserving essential protein function are typically selected. Bacteria can structurally modify their cell wall, membrane, or other targeted components, rendering them less susceptible to treatment (as represented in **Figure 3**) (Belay et al., 2024). For instance, alterations in cell wall architecture can reduce β-lactam binding sites, compromising drug efficacy. Similarly, point mutations affecting ribosomal components can prevent effective binding of antibiotics such as macrolides and tetracyclines, which act by disrupting protein synthesis. These resistance adaptations may result from point mutations, enzymatic modification of target sites, or the use of alternative cellular pathways collectively classified as target site modification, replacement, or bypass (Schaenzer and Wright, 2020).

In Gram-negative bacteria, one of the most common resistance mechanisms involves alterations to the bacterial targets that antibiotics are designed to bind. These modifications typically arise from spontaneous mutations in the genes encoding target proteins and reduce or eliminate drug-binding affinity without compromising the protein’s function. For instance, mutations in the quinolone-resistance-determining regions of DNA gyrase and topoisomerase IV confer resistance to quinolones and fluoroquinolones in both Gram-positive and Gram-negative bacteria (Hooper and Jacoby, 2015). Similarly, resistance to rifampicin in *Mycobacterium tuberculosis* is frequently due to mutations in the rpoB gene, which encodes the β-subunit of RNA polymerase (Goldstein, 2014). Linezolid resistance is often associated with mutations in domain V of the 23S rRNA gene, where multiple mutated alleles correlate with increased MICs. Ribosomal proteins L3 and L4, flanking the linezolid binding site, may also undergo changes that contribute to resistance. Additionally, methylation of the 23S rRNA by enzymes encoded by cfr genes can block the binding of linezolid, chloramphenicol, and clindamycin. This methylation-based resistance is observed across multiple species, including *Staphylococcus*, *Enterococcus*, *E. coli*, and *Bacillus* (Morales et al., 2010; Schaenzer and Wright, 2020). Macrolide resistance is similarly conferred by methylation of the 23S rRNA, particularly at nucleotide A2058, mediated by Erm methyltransferases. Expression of the erm(B) gene results in resistance to macrolides, lincosamides, and streptogramin B, collectively known as the MLSB phenotype (Saderi et al., 2011). In the case of nitrofurantoin, resistance arises mainly through mutations in nfsA and nfsB, which encode nitroreductases responsible for activating the drug. Additional mutations in ribE, encoding lumazine synthase, an enzyme vital for riboflavin biosynthesis, also contribute to resistance (Wan et al., 2021). Resistance also arises from mutations affecting penicillin-binding proteins (such as PBP3 encoded by *ftsI*), which are among the binding sites for carbapenems. They can lower drug affinity and reduce bactericidal activity. These target modifications, often chromosomally mediated, can co-occur with other mechanisms, such as efflux, enhancing resistance complexity (Mourabiti et al., 2025).

In Gram-positive bacteria, target modification likewise constitutes a fundamental resistance mechanism, reducing or eliminating the ability of antibiotics to bind their intended targets, thereby compromising treatment efficacy (Blair et al., 2015). This typically results from genetic mutations or the acquisition of resistance genes that alter key bacterial structures, such as PBPs or ribosomal RNA. β-lactam antibiotics exert their effect by targeting PBPs, enzymes crucial for bacterial cell wall synthesis by forming an irreversible acyl–enzyme complex that blocks transpeptidation, ultimately weakening and lysing the bacterial cell wall. In *Streptococcus pneumoniae*, β-lactam resistance is associated with mosaic pbp genes encoding altered penicillin-binding proteins (PBPs). In *Staphylococcus aureus*, methicillin resistance results from the acquisition of the mecA gene, which encodes PBP2a, a low-affinity variant that enables continued cell wall synthesis despite the presence of β-lactams (Hakenbeck et al., 2012; Navratna et al., 2010; Foster, 2017). Glycopeptide resistance in enterococci arises from the acquisition of van gene clusters that alter the peptidoglycan precursor termini from D-Alanine-D-Alanine to D-Alanine-D-Lactate or D-Alanine-D-Serine, significantly reducing vancomycin binding. Rare but significant instances of vancomycin-resistant *S. aureus* (VRSA) have emerged due to horizontal transfer of vanA from enterococci (Miller et al., 2014). Target bypass also occurs through the acquisition of alternative metabolic enzymes. For example, resistance to trimethoprim and sulfonamides involves acquisition of genes encoding resistant forms of dihydrofolate reductase (DHFR) and dihydropteroate synthase (DHPS), respectively. Additionally, overexpression of DHFR or use of plasmid-borne alternative enzymes can confer resistance by saturating the antibiotic’s inhibitory capacity (Belay et al., 2024).

Resistance also arises from alterations in ribosomal targets. Mutations in the 23S rRNA gene or associated ribosomal proteins disrupt the binding of antibiotics such as macrolides, lincosamides, and streptogramins. These changes impede the drugs’ ability to inhibit protein synthesis and are especially prevalent in clinically significant species like *Staphylococcus aureus* and *Streptococcus pneumoniae* (Gomez et al., 2017).

### 2.4 Enhanced drug efflux: RND-dominated mechanisms in Gram-negatives versus MFS and ABC systems in Gram-positives

**Figure 4:**
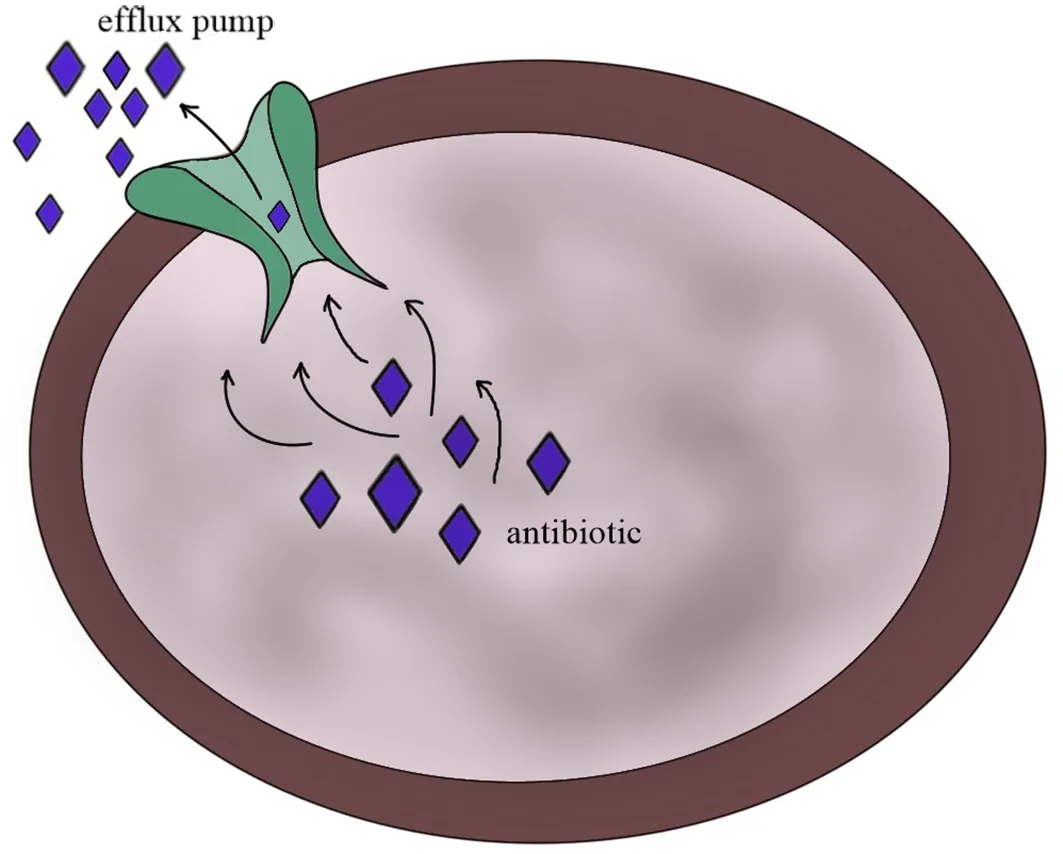
Efflux pumps (adapted from, 55, 58).

Efflux pumps (presented in **Figure 4**) are vital contributors to AMR, facilitating the active extrusion of diverse antibiotic classes (Lorusso et al., 2022). Although efflux-mediated resistance occurs in both Gram-positive and Gram-negative bacteria, the predominant pump families and their clinical significance differ substantially between the two groups (Blair et al., 2015; Munita and Arias, 2016; Lorusso et al., 2022). To date, six main families have been identified: the small multidrug resistance (SMR) family, major facilitator superfamily (MFS), resistance–nodulation–division (RND) family, ATP-binding cassette (ABC) superfamily, multidrug and toxic compound extrusion (MATE) family, and the more recently described proteobacterial antimicrobial compound efflux (PACE) family. These systems are widespread across bacterial species and are increasingly detected in both clinical and environmental isolates. Efflux pumps are categorized by their energy source into primary pumps (ATP-dependent) and secondary pumps (proton motive force-driven).

In Gram-negative bacteria, the RND family is predominant, typically forming a tripartite complex consisting of an outer membrane protein (OMP), a membrane fusion protein (MFP), and an inner membrane transporter (IMP). RND pumps play a significant role in resistance to multiple antibiotics, including tetracyclines, fluoroquinolones, aminoglycosides, and beta-lactams (Webber and Piddock, 2003; Soto, 2013; Sharma et al., 2019; Lorusso et al., 2022). It represents another layer of carbapenem resistance. In *P. aeruginosa*, upregulation of multidrug efflux pumps (e.g., the MexAB-OprM system) can actively expel carbapenems and other antibiotics from the cell, lowering intracellular drug levels and contributing to treatment failure. Regulatory mutations (e.g., in *nalD*, which modulates efflux expression) have been associated with increased efflux and reduced susceptibility (Mourabiti et al., 2025).

Efflux pumps are transport proteins that allow bacteria to survive antibiotic exposure by actively removing antimicrobial agents from the cell, thereby reducing intracellular drug concentration. These systems, present in all living organisms, are encoded by genes located either on the chromosomes or plasmids (Poole, 2007).

Gram-positive bacteria typically uses four of the families: MFS, ABC, SMR, and MATE, while the RND family is specific to Gram-negative species. MFS pumps, such as *NorA* from *Staphylococcus aureus* and *PmrA* from *Streptococcus pneumoniae*, are prominent in Gram-positive bacteria and function through symport, antiport, or uniport mechanisms using transmembrane helices (Quistgaard et al., 2016). ABC transporters, found across all domains of life, use ATP hydrolysis to drive the export of antibiotics and toxins. Each ABC transporter comprises two transmembrane domains and two nucleotide-binding domains, which coordinate to move substances out of the cell (Akhtar and Turner, 2022). The SMR family consists of small proteins that function as homodimers or homotetramers, using the proton motive force in an antiport mechanism, importing protons while expelling antibiotics. MATE transporters, on the other hand, use a sodium ion gradient to expel a broad range of toxic compounds, including antibiotics, through sodium/drug antiport activity. Structurally, they consist of twelve transmembrane helices arranged symmetrically. Collectively, these efflux systems play a critical role in multidrug resistance, particularly in Gram-positive pathogens, by contributing to the active extrusion of antibiotics and reducing the efficacy of antimicrobial treatments (Chetri, 2023). .

Given the structural and functional differences between Gram-positive and Gram-negative bacteria, their predominant resistance mechanisms are comparatively outlined in **Table 2**.

**Table 2:** Comparative overview of principal antibiotic resistance mechanisms in Gram positive and Gram negative bacteria (Donlan, 2002; Webber and Piddock, 2003; Hakenbeck et al., 2012; Kostakioti et al., 2013; Soto, 2013; Banerjee et al., 2015; Rabin et al., 2015; Bonomo, 2017; Gomez et al., 2017; Naas et al., 2017; Rajagopal and Walker, 2017; Armbruster and Parsek, 2018; Bonomo et al., 2018; Egorov et al., 2018; Davin-Regli et al., 2019; De Oliveira et al., 2020; Han et al., 2020; Rather et al., 2021; Lorusso et al., 2022; Intra et al., 2023; Elfadadny et al., 2024; Olaimat et al., 2024; Hu and Chua, 2025; Singh et al., 2025).

Mechanism	Gram-positive bacteria	Gram-negative bacteria	Representative clinical examples
Cell envelope structure influencing resistance	Thick peptidoglycan layer; absence of outer membrane; presence of teichoic acids	Thin peptidoglycan layer; outer membrane with lipopolysaccharide (LPS); periplasmic space	Intrinsic resistance of Gram-negatives to vancomycin due to outer membrane barrier
Enzymatic inactivation of antibiotics	Production of β-lactamases (e.g., penicillinases); aminoglycoside-modifying enzymes (acetyltransferases, phosphotransferases)	Extensive production of β-lactamases (ESBLs, AmpC, KPC, NDM, OXA-type carbapenemases); aminoglycoside-modifying enzymes	MRSA (β-lactamase production); ESBL-producing *Klebsiella pneumoniae*; carbapenem-resistant *Acinetobacter baumannii*
Reduced drug uptake/permeability changes	Less prominent; thickened cell wall (e.g., VISA phenotype); biofilm-associated reduced penetration	Major mechanism; porin loss or modification (e.g., OprD in *Pseudomonas aeruginosa*); decreased outer membrane permeability	Imipenem-resistant *P. aeruginosa* (OprD mutation); altered OmpK35/OmpK36 in *K. pneumoniae*
Target site modification (mutation or gene acquisition)	Altered PBPs (e.g., PBP2a encoded by mecA in MRSA); ribosomal methylation (erm genes); van gene clusters (vanA, vanB)	Mutations in DNA gyrase/topoisomerase IV; altered PBPs; rRNA methylation; lipid A modification (colistin resistance)	MRSA (mecA); VRE (vanA); fluoroquinolone-resistant Enterobacterales
Target bypass or replacement	Acquisition of alternative enzymes (e.g., altered DHFR in trimethoprim resistance); alternative PBPs	Acquisition of plasmid-mediated resistant enzymes (e.g., resistant DHFR, DHPS variants)	Trimethoprim-resistant *Staphylococcus aureus*; sulfonamide-resistant Enterobacterales
Efflux pump overexpression	Mainly MFS, ABC, SMR, and MATE families; typically single-component systems	Prominent RND family pumps forming tripartite systems (OMP–MFP–IMP); broad substrate range	NorA in *S. aureus*; AcrAB-TolC in *E. coli*; MexAB-OprM in *P. aeruginosa*
Biofilm-associated resistance	Frequent in chronic infections; reduced antibiotic penetration; altered metabolic state	Highly significant; biofilm formation on medical devices; combined with efflux and permeability barriers	Device-associated infections by *S. aureus*; catheter-associated infections by *P. aeruginosa*

Reduced drug uptake plays a less prominent role in Gram-positive resistance compared with Gram-negative organisms, as Gram-positive bacteria lack an outer membrane and instead possess a thick but relatively permeable peptidoglycan layer (Yang et al., 2016; Rajagopal and Walker, 2017). Nevertheless, they can limit antimicrobial efficacy through cell wall modifications and biofilm formation. Biofilms are structured microbial communities embedded in a self-produced extracellular matrix that enhances tolerance to antibiotics and host immune defenses. Their development involves sequential stages of attachment, growth, maturation, and dispersion, and is regulated in part by quorum sensing, which coordinates virulence and stress responses. Biofilm-associated infections, including urinary tract infections, endocarditis, osteomyelitis, and device-related infections, are particularly difficult to eradicate and account for a substantial proportion of chronic infections, underscoring the need for innovative therapeutic strategies (Heilmann and Götz, 2009; Blair et al., 2015; Reygaert, 2018; Wang et al., 2024).

Importantly, resistance mechanisms rarely function in isolation as summarized in **Table 3**. In clinically significant pathogens, particularly among ESKAPE organisms, multidrug resistance frequently results from the convergence and synergistic interaction of multiple mechanisms operating simultaneously. For example, enzymatic drug inactivation may coexist with reduced membrane permeability and active efflux, producing cumulative effects that markedly diminish intracellular antibiotic concentrations beyond the contribution of any single determinant (Blair et al., 2015; Breijyeh et al., 2020; Elshobary et al., 2025). Such synergistic resistance enhances survival under antimicrobial pressure and accelerates the emergence of high-level multidrug-resistant phenotypes. However, the acquisition or overexpression of resistance determinants often imposes biological fitness costs, including impaired growth rates, metabolic burden, or reduced competitive capacity in antibiotic-free environments. Under sustained selective pressure, bacteria may acquire compensatory mutations that mitigate these costs, thereby stabilizing resistant lineages and facilitating their persistence and dissemination (Munita and Arias, 2016). Clinically, not all resistance mechanisms exert equivalent impact; a hierarchy exists in which determinants that compromise first-line or last-resort agents, such as carbapenemases in Gram-negative pathogens or vancomycin resistance in enterococci, have disproportionately severe therapeutic and prognostic implications. Recognizing the interplay between mechanistic synergy, evolutionary adaptation, and clinical consequence is essential for prioritizing surveillance, optimizing treatment strategies, and guiding antimicrobial development (Munita and Arias, 2016; De Oliveira et al., 2020; GBD 2021 Antimicrobial Resistance Collaborators, 2024; Sati et al., 2025).

**Table 3:** Antibiotic resistance mechanisms (Webber and Piddock, 2003; Heilmann and Götz, 2009; Hooper and Jacoby, 2015; Munita and Arias, 2016; Gomez et al., 2017; Lorusso et al., 2022).

Resistance mechanism	Description	Typical bacteria
Enzymatic Inactivation	Bacteria produce enzymes that destroy or modify antibiotics (e.g., β-lactamases).	*Enterobacteriaceae*, *Pseudomonas* spp.
Target Modification	Alteration of antibiotic targets prevents drug binding (e.g., mutated PBPs).	MRSA, *Mycobacterium* spp.
Efflux Pumps	Transport proteins actively expel antibiotics from the cell	*Pseudomonas aeruginosa*, *E. coli*.
Biofilm Formation	Structured communities with protective matrix limit antibiotic penetration and action.	*Staphylococcus epidermidis*, *Pseudomonas aeruginosa*.

## 3 ESKAPE pathogens: clinical convergence of Gram-positive and Gram-negative resistance mechanisms

The convergence of enzymatic degradation, structural target alteration, permeability barriers, and multidrug efflux systems is particularly evident among ESKAPE pathogens, in which the accumulation and horizontal dissemination of resistance determinants drive the emergence of highly resilient multidrug-resistant phenotypes (Munita and Arias, 2016; De Oliveira et al., 2020).

Antimicrobial-resistant ESKAPE pathogens: *Enterococcus faecium, Staphylococcus aureus, Klebsiella pneumoniae, Acinetobacter baumannii, Pseudomonas aeruginosa*, and *Enterobacter* spp. pose a significant global threat to public health. The acquisition and dissemination of resistance genes among these organisms have led to limited treatment options for severe infections, an increased disease burden, and elevated mortality rates due to therapeutic failure (De Oliveira et al., 2020). *Enterococcus faecium* has emerged as a major cause of healthcare-associated infections, driven largely by hospital-adapted lineages with increasing resistance to vancomycin. In the United States, its dissemination occurred in two distinct waves: an initial rise in the 1980s associated with extensive third-generation cephalosporin use and the emergence of vancomycin and ampicillin-resistant enterococci, followed by a second wave characterized by the dominance and global spread of vancomycin-resistant *E. faecium* (VREfm). In Europe and Australia, VREfm prevalence among hospitalized patients has risen substantially, with Australia reporting particularly high rates in bloodstream isolates. Most hospital-associated infections are attributed to strains belonging to clonal complex 17 (CC17), a lineage widely distributed in healthcare settings and also detected in animal reservoirs and food sources. Despite its environmental dissemination, community-acquired infections remain relatively uncommon, underscoring the strong association of CC17 with hospital environments (Top et al., 2008; Lee et al., 2019).

Urinary tract infections in hospitalized patients, particularly those with indwelling devices, comorbidities, or prior antibiotic exposure, are increasingly complicated by antimicrobial resistance (Huang et al., 2025). The organism also causes infective endocarditis, especially in patients with prosthetic valves or prior cardiac procedures, where outcomes remain poor despite combination therapy. In addition, it contributes to intra-abdominal, wound, and other healthcare-associated infections, often following gastrointestinal colonization under antibiotic selective pressure. Although predominantly hospital-associated, its growing presence in community-acquired infections highlights the need for continued surveillance and targeted antimicrobial strategies (Sangiorgio et al., 2024; Huang et al., 2025; Radford-Smith and Anthony, 2025).

*Staphylococcus aureus* is a Gram-positive coccus that typically forms clusters resembling grape bunches. It exhibits nonfastidious growth characteristics and is a common constituent of the normal skin and mucosal microbiota, particularly in the anterior nares and perineal regions of humans and various animal hosts. Colonization is widespread in the general population, and transmission occurs primarily through direct contact or, less frequently, via airborne dissemination. Historically, infections caused by *Staphylococcus* spp. were effectively treated with penicillin However, penicillin-resistant, β-lactamase-producing strains emerged shortly after the drug’s introduction in the 1940s, and their prevalence rose rapidly: by 1948, roughly half of hospital isolates were already resistant, rising to approximately 80% by the late 1950s. Currently, the majority of clinical isolates exhibit resistance to penicillin G through β-lactamase production, in both community- and healthcare-associated settings (Santajit and Indrawattana, 2016; Shemetov et al., 2024; Michalik et al., 2025).

Methicillin-resistant S. aureus (MRSA) was first identified in the early 1960s. According to the World Health Organization (WHO) and the Centers for Disease Control and Prevention (CDC), methicillin-resistant *Staphylococcus aureus* (MRSA) has been consistently classified as a high-priority pathogen due to its virulence, resistance profile, and public health impact. The term “hospital-acquired MRSA” (HA-MRSA) is commonly used to describe strains that originate in healthcare environments, where the pathogen was initially identified. Transmission of MRSA primarily occurs through direct contact with infected wounds or contaminated hands. If left untreated, MRSA infections can progress to severe clinical conditions, including pneumonia, bloodstream infections (BSIs), sepsis, and surgical site infections (SSIs). In addition to healthcare-associated MRSA (HA-MRSA), other epidemiological classifications include community-associated MRSA (CA-MRSA), which arises in individuals without recent healthcare exposure, and livestock-associated MRSA (LA-MRSA), which is linked to transmission from animals, particularly in agricultural settings (Konstantinovski et al., 2022; Alghamdi et al., 2023; Siddiqui and Koirala, 2025).

Glycopeptide antibiotics, particularly vancomycin and teicoplanin, are commonly employed as first-line agents in the management of MRSA infections. Nonetheless, the selective pressure exerted by glycopeptide use has facilitated the emergence of strains with reduced susceptibility to vancomycin, referred to as vancomycin-intermediate *S. aureus* (VISA), as well as strains exhibiting full resistance, designated vancomycin-resistant *S. aureus* (VRSA). Most VISA isolates also display diminished susceptibility to teicoplanin, and the term glycopeptide-intermediate *S. aureus* is used to describe such phenotypes. VISA was first reported in Japan in the mid-1990s, and subsequent cases have been documented in Asia, North America, and Europe. VRSA poses an even greater clinical challenge due to the acquisition of the vanA gene from vancomycin-resistant enterococci (VRE) in conjunction with the mecA gene associated with MRSA, thereby conferring resistance to both methicillin and vancomycin and limiting therapeutic options (Blechman and Wright, 2024; Brdová et al., 2024).

*Klebsiella pneumoniae* is a Gram-negative, encapsulated bacillus belonging to the family *Enterobacteriaceae*. It is easily cultivated in laboratory conditions and is frequently implicated in healthcare-associated infections. These infections may originate endogenously or be acquired through direct contact with colonized or infected individuals. In recent years, *K. pneumoniae* has become a significant clinical concern due to its capacity to acquire and express a diverse array of β-lactamase enzymes, which hydrolyze and inactivate β-lactam antibiotics, including penicillins, cephalosporins, and carbapenems. *Klebsiella pneumoniae* frequently acquires resistance to antimicrobial agents through genetic modifications that alter drug targets or neutralize drug efficacy. In clinical practice, β-lactam antibiotics remain the cornerstone of treatment for *K. pneumoniae* infections. These antibiotics exert their bactericidal effect by covalently binding to PBPs, which are essential for the cross-linking of peptidoglycan chains during bacterial cell wall synthesis. In response, *K. pneumoniae* produces β-lactamase enzymes that hydrolyze the β-lactam ring, thereby inactivating the antibiotic and conferring resistance (Li et al., 2023; Huy, 2024). It is associated with some of the highest morbidity and mortality rates in developed countries. The most common predisposing conditions for *K. pneumoniae* infections include diabetes mellitus, cancer, and hepatobiliary disorders. In cases of community-acquired K. pneumoniae bacteremia, diabetes mellitus is frequently reported as a comorbidity. Conversely, nosocomial *K. pneumoniae* bacteremia is often linked to neoplastic diseases, largely due to factors such as extended hospital stays, invasive procedures, chemotherapy, and prior antibiotic exposure (Tsai et al., 2010; Asokan et al., 2025).

*Acinetobacter baumannii* is a Gram-negative member of the ESKAPE group of pathogens and represents a significant global health threat due to its association with severe, predominantly nosocomial infections and high mortality rates. In recent years, *A. baumannii* has demonstrated widespread multidrug resistance (MDR), largely attributed to the overuse of antibiotics and inadequate antimicrobial stewardship. MDR isolates are commonly linked to prolonged hospitalization, the use of invasive devices such as catheters and mechanical ventilation, and infections in immunocompromised or critically ill patients. The advent of next-generation sequencing (NGS) has markedly improved the diagnosis of *A. baumannii* infections, enabling rapid detection of resistance genes and facilitating the implementation of personalized therapeutic strategies. The principal mechanisms of antimicrobial resistance in *A. baumannii* involve reduced permeability or active efflux of antibiotics across the bacterial membrane, target site modification, and enzymatic inactivation of antimicrobial agents (Kyriakidis et al., 2021; Scoffone et al., 2025). *Acinetobacter baumannii* is highly adept at adhering to and colonizing various surfaces. It can withstand both dry and moist conditions, as well as exposure to common disinfectants and ultraviolet light (Shi et al., 2024).

*Pseudomonas aeruginosa* is an opportunistic Gram-negative pathogen and a major contributor to morbidity and mortality, particularly among patients with cystic fibrosis and those who are immunocompromised (Pang et al., 2019). The successful eradication of *P. aeruginosa* infections has become increasingly challenging due to the organism’s exceptional ability to evade antimicrobial agents. This resistance is mediated through a combination of intrinsic and acquired mechanisms that collectively render most conventional antibiotics ineffective. Additionally, *P. aeruginosa* exhibits adaptive resistance, a dynamic and reversible form of resistance that includes biofilm-associated protection and the generation of multidrug-tolerant persister cells. These adaptive mechanisms play a critical role in the persistence, treatment failure, and recurrence of chronic infections (Pang et al., 2019; Elfadadny et al., 2024). *Pseudomonas aeruginosa* is associated with a variety of severe infections, including ventilator-associated pneumonia (VAP), surgical site infections, urinary tract infections (UTIs), and bloodstream infections (BSIs). UTIs caused by this pathogen are especially problematic in healthcare settings, often linked to catheter use, structural urinary tract abnormalities, or immunocompromised states. Its capacity for biofilm formation, invasion of bladder epithelial cells, and other intrinsic and acquired resistance mechanisms significantly complicates treatment. Notably, *P. aeruginosa* is a leading cause of catheter-associated urinary tract infections (CAUTIs), where biofilms on catheter surfaces make eradication particularly difficult (Hu and Chua, 2025).

*Enterobacter* species are Gram-negative, rod-shaped, opportunistic pathogens belonging to the family *Enterobacteriaceae*. They are commonly implicated in severe healthcare-associated infections, including urinary tract infections (UTIs), septicemia, and pneumonia. These infections predominantly occur in vulnerable populations such as patients in intensive care units (ICUs) receiving mechanical ventilation, premature neonates, individuals with diabetes or leukemia, trauma patients, and those undergoing immunosuppressive therapy. The genus *Enterobacter* comprises 22 recognized species of Gram-negative bacteria. Among them, two species, *Enterobacter aerogenes* (recently reclassified as *Klebsiella aerogenes*) and *Enterobacter cloacae*, are responsible for the majority of human infections. These opportunistic pathogens are commonly associated with healthcare-associated infections, particularly in immunocompromised individuals and hospitalized patients. *Enterobacter* spp. exhibit intrinsic resistance to ampicillin and many broad-spectrum cephalosporins. Furthermore, through the acquisition of mobile genetic elements, these organisms have developed resistance to multiple additional antibiotics, including third-generation cephalosporins and carbapenems. This multidrug resistance significantly complicates the selection of effective therapeutic options. The rising prevalence of antimicrobial resistance among *Enterobacter* strains has been associated with increased mortality rates, prolonged hospital stays, and substantially higher healthcare costs (Davin-Regli et al., 2019; Intra et al., 2023; Singh et al., 2025).

Critically, the resistance determinants described above do not contribute equally to the global clinical burden, nor are they uniformly distributed across regions and species. From a therapeutic standpoint, mechanisms that compromise last-resort agents carry disproportionate weight: carbapenemase production in Enterobacterales and Acinetobacter, together with emerging plasmid-mediated colistin resistance, represents the most consequential threat, whereas determinants affecting first-line agents are more readily circumvented. Epidemiologically, the dominant mechanisms vary markedly by geography. Among carbapenem-resistant Enterobacterales, NDM has become the most prevalent carbapenemase globally, followed by OXA-48-like and KPC enzymes, yet the predominant type differs by setting: KPC prevails in the Americas and parts of southern Europe, OXA-48-like across the Mediterranean and South Asia, and NDM throughout the Indian subcontinent and, increasingly, worldwide (Bonomo et al., 2018; Han et al., 2020). The literature is not fully concordant on the relative importance of individual mechanisms: estimates of the contribution of efflux overexpression versus enzymatic or porin-mediated resistance differ considerably between *in vitro* and clinical studies, and genotype–phenotype correlation remains imperfect, particularly where resistance arises from regulatory mutations or the combinatorial interaction of multiple low-level determinants. These discrepancies expose a persistent knowledge gap: current surveillance detects the presence of resistance genes far more reliably than their expression or clinical impact, and the dynamics by which mobile genetic elements assemble multidrug-resistant lineages remain incompletely understood. Closing this gap will require harmonized genotype–phenotype datasets and prospective, region-stratified surveillance to translate mechanistic knowledge into clinically actionable priorities (Blair et al., 2015). In Gram-positive pathogens, analogous surveillance gaps exist: the clinical contribution of *cfr*-mediated oxazolidinone resistance versus chromosomal 23S rRNA mutations remains debated, and the dynamics of *vanA* transfer from enterococci to *Staphylococcus aureus*, though rare, represent an under-monitored threat with potentially catastrophic therapeutic consequences. A truly integrated understanding of AMR therefore requires parallel, organism-group-aware surveillance frameworks that account for the mechanistically distinct but clinically convergent resistance landscapes of Gram-positive and Gram-negative pathogens (Miller et al., 2014; Munita and Arias, 2016; Foster, 2017; Gomez et al., 2017).

The comparative table (see **Table 4**) summarizes the principal genetic drivers, prevailing resistance mechanisms, and corresponding therapeutic implications for each ESKAPE pathogen, thereby highlighting mechanistic differentiation and its translational relevance.

**Table 4:** Mechanistic and pharmacological differentiation of ESKAPE pathogens (Tsai et al., 2010; Hakenbeck et al., 2012; Goldstein, 2014; Miller et al., 2014; Foster, 2017; Davin-Regli et al., 2019; Kyriakidis et al., 2021; Wan et al., 2021; Konstantinovski et al., 2022; Intra et al., 2023; Li et al., 2023; Brdová et al., 2024; Huy, 2024; Shi et al., 2024; Asokan et al., 2025; Hu and Chua, 2025).

Pathogen	Key genetic determinants	Dominant resistance mechanisms	PK/PD & therapeutic implications
Enterococcus faecium	*vanA*, *vanB*, altered *pbp5*, aminoglycoside-modifying enzymes	Target modification (D-Ala-D-Lac), reduced β-lactam affinity, high-level aminoglycoside resistance	Loss of vancomycin efficacy; limited bactericidal synergy with β-lactam–aminoglycoside combinations; reliance on linezolid or high-dose daptomycin
Staphylococcus aureus	*mecA*, *mecC*, 23S rRNA mutations, *cfr*	PBP2a-mediated β-lactam resistance, ribosomal modification, biofilm formation	Reduced β-lactam binding (MRSA); need for optimized vancomycin AUC/MIC targets; alternative agents (linezolid, daptomycin) in invasive disease
Klebsiella pneumoniae	*KPC*, *NDM*, *OXA-48*, ESBL genes, porin mutations (*ompK35/36*)	Carbapenemase production, β-lactam hydrolysis, permeability reduction	Carbapenem failure at standard exposures; need for novel β-lactam/β-lactamase inhibitors; importance of achieving adequate fT>MIC
Acinetobacter baumannii	OXA-type carbapenemases, efflux overexpression, porin loss	Enzymatic carbapenem degradation, reduced drug penetration	Frequent multidrug resistance; reliance on high-dose regimens, combination therapy, or polymyxins despite toxicity concerns
Pseudomonas aeruginosa	*AmpC*, *MexAB-OprM*, *oprD* mutations	Inducible AmpC production, RND efflux, carbapenem entry loss	Narrow therapeutic window; PK/PD optimization critical; resistance selection with subtherapeutic exposure
Enterobacter spp.	Chromosomal *AmpC*, ESBLs, carbapenemases	Inducible cephalosporin resistance, β-lactamase-mediated hydrolysis	Risk of resistance emergence during therapy; carbapenem or advanced inhibitor combinations often required

While the individual resistance mechanisms described above can each independently compromise antibiotic efficacy, their clinical impact is substantially amplified when bacteria adopt a biofilm lifestyle. Among ESKAPE pathogens, biofilm formation represents a critical additional layer of resistance that operates independently of, yet synergistically with, the molecular mechanisms discussed in preceding sections (Kostakioti et al., 2013; Pang et al., 2019; De Oliveira et al., 2020).

## 4 Biofilm-associated resistance: organism-specific matrix architecture and therapeutic implications in Gram-positive and Gram-negative pathogens

Biofilm formation represents an adaptive lifestyle in which microorganisms transition from a free-living, single-cellular state to a coordinated multicellular community, enhancing survival across a variety of environmental conditions (Kostakioti et al., 2013). While biofilm development follows broadly conserved stages across bacterial species, its molecular execution, matrix composition, and contribution to antibiotic tolerance differ substantially between Gram-positive and Gram-negative pathogens (Kostakioti et al., 2013; Munita and Arias, 2016; De Oliveira et al., 2020). These structured microbial communities develop on both biotic and abiotic surfaces, including natural ecosystems and clinical environments. In healthcare settings, biofilms frequently form on ventilators, catheters, and other medical devices, serving as persistent reservoirs for pathogenic organisms and facilitating their transmission to vulnerable patients. Within the host, biofilms provide a protective niche that enables pathogens to evade innate immune responses and antimicrobial therapies, contributing to chronic infections and prolonged colonization (Donlan, 2002; Kostakioti et al., 2013).

Biofilms exhibit a wide range of pathological manifestations and are ubiquitous, colonizing medical implants, living tissues, water systems, industrial surfaces, hospital environments, and numerous other biotic and abiotic substrates. Microorganisms within biofilms undergo significant phenotypic and transcriptional changes, including altered gene expression profiles, reduced metabolic activity and growth rates, increased production of virulence factors, and a marked resistance to conventional antibiotics. These adaptations contribute to the persistence of biofilm-associated infections and their resilience in both clinical and environmental settings (Rather et al., 2021). Biofilm formation begins with the initial adhesion of free-living microorganisms to a surface, marking a critical transition from a dispersed to a structured community lifestyle. In the early stages, microbial cells are loosely and reversibly attached, often exhibiting polar adherence to the surface. As the process progresses, cells reorient to lie flat and establish irreversible attachment, a phase that confers increased stability and initiates the development of resistance to various physical and environmental factors that would otherwise impede biofilm maturation (Banerjee et al., 2015; Olaimat et al., 2024).

Bis-(3′–5′)-cyclic dimeric guanosine monophosphate (c-di-GMP) is a key intracellular second messenger that regulates early biofilm formation. Surface contact activates membrane-associated sensing systems such as Pil-Chp, leading to increased intracellular c-di-GMP levels. Elevated c-di-GMP suppresses flagellar motility and stimulates extracellular matrix production, driving the transition from reversible attachment to stable, irreversible biofilm establishment (Armbruster and Parsek, 2018). Following successful surface adhesion, microorganisms begin to proliferate and aggregate within a self-produced extracellular polymeric substance (EPS), leading to the formation of microcolonies. This process is facilitated by elevated intracellular concentrations of c-di-GMP, which promote matrix production and suppress motility. Flagella-mediated motility plays a critical role in the initial surface interactions, while type IV pili contribute to intercellular interactions and aggregation, both of which are essential for the structural development of microcolonies during early biofilm maturation (Rabin et al., 2015). The extracellular polymeric substance (EPS) is central to biofilm maturation, mediating surface adhesion, structural stability, microbial aggregation, and protection against immune defenses, antimicrobial agents, and environmental stressors. It also functions as a reservoir for signaling molecules, enzymes, and metabolic by-products that enhance biofilm resilience. As maturation progresses, biofilms develop complex, spatially organized architectures with metabolically stratified layers. The lifecycle culminates in the dispersal phase, during which cells detach either actively or passively to colonize new niches (Otto, 2013; Flemming et al., 2016; Toyofuku et al., 2016).

Beyond structural protection, biofilm architecture profoundly alters antimicrobial susceptibility and therapeutic response (Flemming et al., 2016). Biofilm-associated infections are notoriously difficult to eradicate because biofilm growth promotes antibiotic tolerance that is mechanistically distinct from inherited resistance. Within biofilms, reduced antimicrobial penetration through the matrix, spatial gradients in oxygen and nutrients, and heterogeneous metabolic states collectively decrease antibiotic killing, particularly for agents whose activity depends on active growth. Consequently, conventional susceptibility testing based on planktonic cells can underestimate exposure requirements in biofilm infections, contributing to an apparent “MIC inflation” *in vivo* and helping explain therapeutic failure despite *in vitro* susceptibility. Biofilms also enrich persister cells, a transient subpopulation characterized by slowed metabolism and stress-adapted physiology that can survive bactericidal antibiotic concentrations without stable genetic resistance, thereby driving relapse once treatment is stopped. These features motivate anti-biofilm pharmacological strategies that complement conventional antibiotics, including approaches that disrupt or degrade matrix components, interfere with regulatory/signaling pathways involved in biofilm maintenance, and use combination regimens to enhance penetration and killing of both metabolically active and dormant subpopulations (Costerton et al., 1999; Kostakioti et al., 2013; Soto, 2013; Flemming et al., 2016; Sauer et al., 2022).

Active dispersion is driven by microbial motility and the enzymatic degradation of EPS, while passive dispersion is influenced by external physical forces, such as fluid shear stress. Several factors contribute to the initiation of dispersal, including overpopulation, nutrient depletion, increased competition, and environmental stressors such as temperature fluctuations, oxygen limitation, and the accumulation of metabolic by-products. On a molecular level, dispersal is associated with the upregulation of genes involved in motility and matrix degradation, and the downregulation of genes responsible for polysaccharide production and fimbriae synthesis. These regulatory shifts enable cells to detach from the biofilm matrix and colonize new surfaces, thereby initiating a new cycle of biofilm development (Costerton et al., 1999; Sauer et al., 2022). Biofilm-associated growth further limits antibiotic efficacy, creating phenotypic tolerance that complements genetic resistance mechanisms and reinforces the need for alternative therapeutic strategies, such as lasso peptides (Costerton et al., 1999; O’Neill, 2016; Sauer et al., 2022).

While the stages of biofilm development described above are broadly conserved, their molecular execution differs substantially between Gram-positive and Gram-negative organisms, reflecting their distinct envelope architectures. In Gram-positive bacteria such as *Staphylococcus aureus*, biofilm matrices are frequently dominated by polysaccharide intercellular adhesin (PIA), synthesized via the *icaADBC* operon, together with surface-anchored adhesins that mediate initial attachment and detachment-regulating mechanisms (Otto, 2013). In Gram-negative bacteria, by contrast, exopolysaccharide composition is more diverse and organism-specific: *Pseudomonas aeruginosa* relies on a combination of Psl, Pel, and alginate exopolysaccharides with distinct chemical structures and charge properties (Franklin et al., 2011), while outer-membrane vesicles contribute additional structural and signaling functions largely absent from Gram-positive biofilms (Schwechheimer and Kuehn, 2015). These structural differences also carry clinical implications, as anti-biofilm strategies targeting matrix composition may require organism-specific design rather than a uniform strategy across Gram-positive and Gram-negative pathogens (Kostakioti et al., 2013; Koo et al., 2017).

Despite the well-characterized stages of biofilm development, several aspects of biofilm-associated resistance remain contested and clinically underexplored. A central unresolved issue is the discordance between *in vitro* susceptibility and *in vivo* therapeutic response: conventional susceptibility testing performed on planktonic cells systematically underestimates the antibiotic exposure required to eradicate biofilm-embedded populations, contributing to treatment failure despite apparent *in vitro* susceptibility. Although biofilm-specific endpoints such as the minimum biofilm inhibitory concentration (MBIC) and minimum biofilm eradication concentration (MBEC) have been proposed, the lack of standardized methods, parameters, and clinical breakpoints, endorsed by official agencies such as EUCAST or CLSI, currently prevents their routine clinical implementation (Macià et al., 2014). Compounding this limitation, *in vitro* biofilm models often poorly represent the *in vivo* microenvironment, such that biofilm susceptibility data may not predict *in vivo* anti-biofilm activity any better than conventional planktonic testing (Coenye, 2023). The inconsistent use of biofilm endpoints across studies has further generated contradictory findings among anti-biofilm investigations and clinical trials (Thieme et al., 2019). Moreover, the relative contributions of matrix-limited drug penetration, metabolic dormancy, and persister-cell formation to overall biofilm tolerance remain debated and likely vary by organism and infection site. While numerous anti-biofilm strategies (matrix-degrading enzymes, quorum-sensing inhibitors, and combination regimens) have shown promise experimentally, robust clinical validation is largely lacking. Addressing these gaps will require standardized, clinically validated biofilm susceptibility assays and a deeper mechanistic understanding of biofilm tolerance before anti-biofilm approaches can be reliably integrated into therapeutic guidelines (Hall and Mah, 2017; Koo et al., 2017).

The complexity and organism-specificity of resistance mechanisms, whether enzymatic, structural, or biofilm-associated, underscore the critical importance of accurate and timely detection. The following section examines current and emerging diagnostic approaches for AMR identification, with attention to their differential performance across Gram-positive and Gram-negative organisms (Kostakioti et al., 2013; Koo et al., 2017).

## 5 Diagnostic approaches for AMR detection: differential applicability across Gram-positive and Gram-negative organisms

Rapid and accurate detection of AMR is essential for optimizing therapy and limiting resistance selection pressure. The choice of diagnostic approach and its predictive value are critically influenced by whether the target pathogen is Gram-positive or Gram-negative, as the dominant resistance determinants and their genetic basis differ fundamentally between the two groups (Munita and Arias, 2016; De Oliveira et al., 2020; van Belkum et al., 2020). Conventional culture-based antimicrobial susceptibility testing (AST) remains the gold standard but typically requires 24–72 hours to yield results, often necessitating empiric broad-spectrum therapy and increasing selective pressure. Molecular diagnostic platforms, including PCR-based assays targeting resistance determinants such as carbapenemases and mecA, significantly reduce time-to-result; however, their targeted design limits detection to known resistance genes and may not capture complex phenotypic mechanisms. Emerging genomic approaches provide comprehensive resistance profiling and epidemiological insight, yet their clinical implementation is constrained by cost, infrastructure requirements, and genotype-phenotype discordance, particularly in organisms where resistance involves regulatory mutations, efflux overexpression, or permeability alterations (Ellington et al., 2017; van Belkum et al., 2020).

The growing integration of artificial intelligence and machine-learning-based susceptibility prediction models offers potential for accelerating resistance detection and improving precision therapy; however, these systems require large validated datasets and standardized clinical integration. Artificial intelligence (AI)-assisted susceptibility prediction tools are increasingly being explored to bridge this gap. Machine-learning models trained on genomic and phenotypic datasets show promise in predicting antimicrobial susceptibility patterns and reducing diagnostic latency. Nevertheless, these systems require large, curated datasets, standardized validation, regulatory oversight, and integration into clinical workflows before widespread adoption is feasible (Nguyen et al., 2019; Ren et al., 2022; Luchian et al., 2025).

Economic considerations further complicate implementation. While rapid molecular tests may reduce downstream healthcare costs by enabling earlier targeted therapy, their upfront expense and equipment requirements may limit accessibility in resource-constrained settings. Similarly, advanced sequencing technologies remain cost-prohibitive for many institutions despite declining sequencing costs. Collectively, optimizing resistance diagnostics requires balancing speed, comprehensiveness, economic feasibility, and clinical applicability. A mechanistically informed diagnostic strategy that integrates rapid detection with pharmacodynamic optimization may ultimately reduce resistance selection and improve therapeutic precision (Craig, 1998; Dadgostar, 2019).

Taken together, these diagnostic approaches differ substantially in their clinical impact and readiness for implementation. From a stewardship perspective, rapid phenotypic and molecular assays that shorten time-to-appropriate-therapy currently offer the greatest immediate benefit, whereas genomic and AI-based platforms remain largely confined to reference laboratories and surveillance networks (Nguyen et al., 2019; van Belkum et al., 2020; Ren et al., 2022). The literature is not fully concordant regarding the comparative performance of these methods: studies evaluating molecular versus phenotypic AST report variable sensitivity and turnaround-time gains depending on organism, specimen type, and local epidemiology, and genotype-phenotype discordance rates differ considerably across settings (van Belkum et al., 2020; Ren et al., 2022). Globally, PCR-based detection of carbapenemase and mecA genes remains the most widely implemented rapid molecular approach in routine diagnostics, while comprehensive genomic profiling remains comparatively underused outside specialized centers (van Belkum et al., 2020). A persistent knowledge gap concerns the lack of harmonized, clinically validated thresholds for translating molecular or AI-derived resistance predictions into actionable treatment decisions, and prospective studies directly comparing diagnostic strategies on patient-relevant outcomes remain scarce (Nguyen et al., 2019; van Belkum et al., 2020; Ren et al., 2022).

Beyond their analytical performance, rapid molecular diagnostics have demonstrated measurable downstream clinical benefits. A systematic review and meta-analysis of 31 studies (n=5,920 patients) found that molecular rapid diagnostic testing for bloodstream infections, particularly when paired with antimicrobial stewardship programs, was associated with significantly reduced mortality risk, shorter time to effective therapy, and reduced hospital length of stay (Timbrook et al., 2017). Earlier pathogen and resistance-gene identification also facilitates timely antibiotic de-escalation, thereby reducing unnecessary broad-spectrum antibiotic exposure and supporting stewardship objectives (Timbrook et al., 2017; Wang et al., 2025).

### 5.1 Phenotypic methods

**Figure 5:**
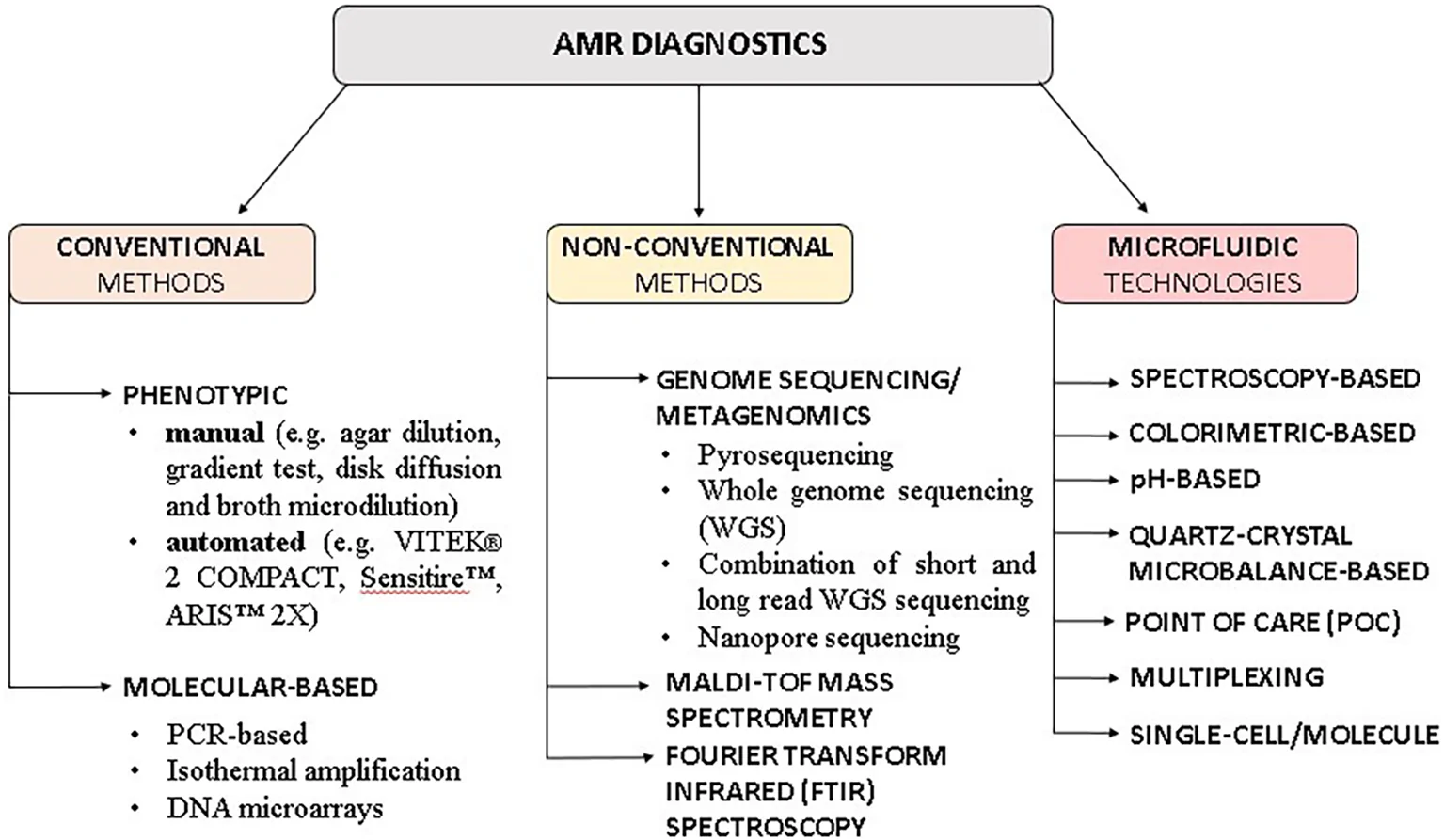
Methods of identification (adapted from 112, 121).

Culture-based methods detect phenotypic resistance by assessing bacterial growth in the presence of antibiotics and can be manual or automated. Manual methods include agar dilution, broth microdilution, disk diffusion, and gradient tests, while automated platforms like VITEK^®^ 2 COMPACT, Sensititre™ ARIS™ 2X, and Alfred 60AST use similar principles, often with ready-made plates or cartridges. These systems provide real-time growth monitoring MIC analysis, offering both qualitative and quantitative results. Disk diffusion measures zones of inhibition, and gradient tests like E-tests are especially useful for fastidious organisms. Interpretation standards are provided by organizations such as EUCAST and CLSI, though discrepancies can exist, for example, the susceptibility breakpoint for amikacin-resistant *Escherichia coli* differs between EUCAST (≤8 mg/L) and CLSI (≤16 mg/L) (Andrews, 2001; Kahlmeter and Brown, 2010; CLSI, 2024; EUCAST, 2024). **Figure 5** presents an overview of the different identification methods.

Phenotypic susceptibility patterns also differ structurally between the two groups: in Gram-negative bacteria, outer-membrane permeability and porin status can confound phenotypic MIC interpretation independent of intrinsic enzymatic resistance, whereas in Gram-positive organisms, phenotypic resistance more directly reflects target-site alterations such as PBP2a-mediated methicillin resistance, without an analogous outer permeability barrier (Munita and Arias, 2016; Rajagopal and Walker, 2017).

While phenotypic methods remain the clinical standard, their inherent limitations in speed and mechanistic resolution have driven the development of molecular-based approaches that directly target resistance determinants at the genetic level (Andrews, 2001; Munita and Arias, 2016).

### 5.2 Molecular-based methods

Molecular-based assays for detecting antibiotic resistance genes (ARGs) offer several advantages over phenotypic methods, including multiplex targeting, precise characterization, and the ability to use non-purified polymicrobial samples. They also allow rapid adaptation to newly emerged resistance factors. However, these methods cannot determine minimum inhibitory concentrations (MICs) and may miss ARGs that are unknown or not specifically targeted. The diversity of resistance genes and the associated costs can also limit their widespread use. Despite these challenges, advancements in nucleic acid amplification, hybridization techniques, and next-generation sequencing have made molecular methods increasingly fast, sensitive, and valuable for routine diagnostics. Techniques such as PCR, isothermal amplification, and DNA microarrays are commonly used for ARG detection and expression analysis (Kaprou et al., 2021; Alsuof et al., 2025).

The clinical utility of ARG-targeted molecular panels also varies by organism group: in Gram-negative bacteria, panels increasingly prioritize carbapenemase genes such as *bla*KPC, *bla*NDM, and *bla*OXA-48-like given their high outbreak potential and plasmid-mediated spread, whereas in Gram-positive bacteria, *mecA*/*mecC* and *vanA*/*vanB* remain the principal molecular targets for MRSA and VRE screening, respectively (Miller et al., 2014; Foster, 2017; Mourabiti et al., 2025).

Fourier transform infrared spectroscopy (FTIR) is an analytical technique based on the absorption of infrared radiation. It can be used to characterize the biomolecular profiles of bacterial cells, allowing differentiation between species. Since each bacterial species and even individual strains have distinct molecular compositions, they produce unique FTIR spectra (Zarnowiec et al., 2015). The FTIR spectrum of a molecule is highly specific, reflecting dense chemical information. Advanced data processing algorithms are required to detect subtle spectral differences, enabling rapid and safe identification of bacterial resistance (Salman et al., 2017; Ciloglu et al., 2021). FTIR-based discrimination has shown particular promise in Gram-positive bacteria for distinguishing methicillin-resistant from methicillin-susceptible *Staphylococcus aureus* (Silvola et al., 2026). In Gram-negative bacteria it has been applied as a first-line typing tool for outbreak investigation of extended-spectrum β-lactamase- and carbapenemase-producing *Klebsiella pneumoniae* (Wang-Wang et al., 2022).

Whole-genome sequencing (WGS) offers the highest resolution for microbial analysis, encompassing all genes within a genome. It enables microbial identification, phylogenetic analysis, mutation detection, discovery of novel genes, retrospective studies, and prediction of phenotypic antibiotic susceptibility. WGS also provides critical insights into antibiotic resistance traits and their mobility, making it invaluable for outbreak investigations and real-time surveillance of antibiotic resistance gene dissemination (Imchen et al., 2020). Whole-genome sequencing is limited by its need for pure cultures, leaving many microbes uncharacterized. Metagenomics overcomes this by sequencing total environmental DNA, allowing identification of both cultured and uncultured microbes, predicting functional potential, and reconstructing whole genomes. It also enables analysis of species interactions, global data sharing, and implementation in low-resource settings. Advances in shotgun metagenomics have already uncovered novel antibiotic resistance genes, demonstrating their potential for large-scale ARG surveillance and environmental monitoring (Su et al., 2017; Steen et al., 2019). WGS-based surveillance has proven particularly valuable for tracking the global spread of Gram-negative carbapenemase genes, such as *bla*KPC and *bla*NDM, across plasmid lineages and high-risk clones in *Klebsiella pneumoniae* and other Enterobacterales, while in Gram-positive pathogens it is increasingly used to resolve clonal transmission of MRSA and VRE within healthcare settings (Teo et al., 2022).

To facilitate comparison between phenotypic, molecular, and emerging diagnostic methods, a summary of their underlying principles, strengths, limitations, and applications in AMR surveillance and stewardship is provided in **Table 5**.

**Table 5:** Methods of identification – (Andrews, 2001; Zhao and Drlica, 2001; Drusano, 2004; Drlica and Zhao, 2007; Nikaido, 2009; Kahlmeter and Brown, 2010; Ehmann et al., 2012; Lewis, 2013; Hecker et al., 2015; Salman et al., 2017; Su et al., 2017; Wenning and Theiler, 2018; Steen et al., 2019; Imchen et al., 2020; Ciloglu et al., 2021; Kaprou et al., 2021; Teo et al., 2022; Wang-Wang et al., 2022; CLSI, 2024; EUCAST, 2024; World Health Organization, 2024; Alsuof et al., 2025; Wang et al., 2025; Silvola et al., 2026).

Diagnostic approach	Principle	Main advantages	Key limitations	Role in AMR surveillance & stewardship
Disk Diffusion (Kirby–Bauer)	Measurement of inhibition zones around antibiotic disks	Low cost; simple; standardized (EUCAST/CLSI); widely available	Qualitative; longer turnaround time (18–24 h); limited resolution	Routine clinical decision-making; baseline resistance monitoring
Broth/Agar Dilution (MIC determination)	Determination of minimum inhibitory concentration (MIC)	Quantitative results; gold standard; precise susceptibility categorization	Labor-intensive (manual methods); requires viable culture; slower results	Guides targeted therapy; supports breakpoint-based stewardship decisions
Automated Phenotypic Systems (e.g., VITEK^®^, Sensititre™)	Automated growth monitoring and MIC calculation	Faster turnaround; standardized; high throughput	Expensive; limited panels; possible discrepancies with reference methods	Hospital surveillance; rapid optimization of empirical therapy
PCR (Targeted gene detection)	Amplification of specific resistance genes	Rapid; high sensitivity/specificity; detects known ARGs	Cannot detect unknown genes; does not measure MIC; may not reflect phenotypic expression	Early detection of high-risk resistance (e.g., mecA, vanA, carbapenemases); outbreak control
Multiplex PCR/Isothermal Amplification	Simultaneous amplification of multiple targets	Rapid; detects multiple ARGs in a single assay	Limited to predefined targets; cost considerations	Screening of multidrug-resistant organisms (MDROs)
DNA Microarrays	Hybridization-based detection of multiple resistance genes	High multiplexing capacity; broad ARG profiling	Expensive; requires technical expertise; limited dynamic adaptability	Large-scale epidemiological surveillance
Whole-Genome Sequencing (WGS)	Complete genome sequencing and bioinformatic analysis	Highest resolution; detects known and novel ARGs; tracks transmission; outbreak investigation	Cost; bioinformatics expertise required; slower than targeted PCR; requires pure culture	High-resolution surveillance; epidemiological tracking; resistance prediction
Metagenomics (Shotgun sequencing)	Sequencing total DNA from clinical/environmental samples	Detects unculturable organisms; comprehensive resistome analysis	High cost; complex data analysis; limited standardization	Environmental surveillance; One Health AMR monitoring
MALDI-TOF MS	Mass spectrometry-based protein profiling	Rapid identification; minimal sample preparation	Primarily for identification; limited direct resistance detection (except specific markers)	Accelerates organism identification → earlier targeted therapy
FTIR Spectroscopy	Infrared spectral fingerprinting of biomolecules	Rapid; non-destructive; low consumable cost	Requires advanced algorithms; limited standardization	Emerging tool for rapid resistance screening
Flow Cytometry/Microcalorimetry	Detection of metabolic or growth changes in real time	Rapid phenotypic detection; potential for early AST	Still under development; specialized equipment	Future rapid AST to reduce inappropriate empirical therapy

## 6 PK/PD optimization as a resistance-prevention strategy: organism-group-specific considerations

Antimicrobial efficacy is determined by pharmacokinetic/pharmacodynamic (PK/PD) relationships that link drug exposure to bacterial killing. Three principal indices guide antibacterial activity: the time that free drug concentrations remain above the MIC (fT>MIC) for time-dependent agents such as β-lactams, the peak-to-MIC ratio (Cmax/MIC) for concentration-dependent drugs like aminoglycosides, and the AUC/MIC ratio for agents including fluoroquinolones and vancomycin. Failure to achieve optimal PK/PD targets not only compromises efficacy but also promotes resistance emergence (Craig, 1998; Zhao and Drlica, 2001; Drusano, 2004).

The mutant selection window (MSW) concept further explains how subtherapeutic exposures select resistant subpopulations (Zhao and Drlica, 2001). When antibiotic concentrations remain between the MIC and the mutant prevention concentration (MPC), first-step resistant mutants may be enriched. Maintaining exposures above the MPC, where feasible, can help suppress resistance development (Drlica and Zhao, 2007).

The evolution of β-lactamase inhibitors exemplifies translational adaptation to resistance. While early agents such as clavulanic acid targeted narrow-spectrum enzymes, the rise of ESBLs and carbapenemases prompted development of structurally novel inhibitors such as avibactam and vaborbactam. Nevertheless, resistance to these next-generation inhibitors has already emerged, highlighting the ongoing interplay between antimicrobial innovation and bacterial evolution (Bush and Jacoby, 2010; Ehmann et al., 2012; Hecker et al., 2015).

A detailed molecular characterization of resistance pathways has become central to contemporary antibiotic development. Modern strategies integrate structure-based design targeting conserved or previously underexploited sites, improved membrane penetration in Gram-negative bacteria, efflux avoidance or inhibition, and rational combination therapies to enhance exposure and suppress resistance. In parallel, PK/PD modeling, hollow-fiber infection systems, and translational *in vivo* models are increasingly used to define resistance-suppressive dosing regimens prior to clinical evaluation. The integration of molecular resistance biology with pharmacodynamic optimization is therefore essential for advancing next-generation antimicrobials and disrupting the cycle of resistance emergence (Drusano, 2004; Lewis, 2013; Hecker et al., 2015).

The clinical relevance of PK/PD-guided strategies varies considerably depending on the antibiotic class and resistance mechanism involved. Optimizing fT>MIC for β-lactams against Enterobacterales is now well supported by clinical evidence (Craig, 1998; Drusano, 2004), whereas the translational value of MSW/MPC-based dosing for preventing resistance emergence remains more contested, with conflicting results between *in vitro* hollow-fiber models and clinical outcomes (Zhao and Drlica, 2001; Drlica and Zhao, 2007). Globally, the introduction of novel β-lactamase inhibitors such as avibactam and vaborbactam has had the greatest impact on extending the utility of existing β-lactams against carbapenemase-producing organisms, though resistance to these agents is already emerging in some regions (Bush and Jacoby, 2010; Ehmann et al., 2012; Hecker et al., 2015). A major knowledge gap remains the limited integration of PK/PD principles into the design and regulatory evaluation of new antimicrobials, as well as insufficient data on how resistance-suppressive dosing regimens perform in heterogeneous clinical populations, including pediatric patients (Craig, 1998; Nikaido, 2009; Lewis, 2013).

While the diagnostic methods discussed above address the detection of existing resistance, PK/PD optimization addresses a complementary objective: preventing the *de novo* emergence of resistance through appropriately dosed therapy rather than identifying resistance once it has occurred. The clinical relevance of PK/PD targets also differs by organism group. In Gram-negative bacteria, fT>MIC optimization for β-lactams is particularly critical in infections involving inducible AmpC production or RND efflux-mediated resistance, such as those caused by *Pseudomonas aeruginosa* and *Enterobacter* spp., where subtherapeutic exposure readily selects for derepressed AmpC mutants or efflux-overexpressing variants (Davin-Regli et al., 2019; Pang et al., 2019). In Gram-positive pathogens such as MRSA, PK/PD considerations more often guide glycopeptide or oxazolidinone dosing, where insufficient exposure can favor the emergence of target-site mutations (e.g., in *vanA* or 23S rRNA) rather than enzymatic or efflux-based escape (Miller et al., 2014; Foster, 2017).

The mechanistic insights derived from PK/PD optimization, particularly the recognition that subtherapeutic exposure selects for resistance at conserved target sites, highlight the urgent need for antimicrobial agents that engage novel bacterial targets. Lariocidin, a ribosome-targeting lasso peptide, exemplifies this principle by exploiting a previously unexploited ribosomal site to circumvent resistance mechanisms operating across both Gram-positive and Gram-negative pathogens (Craig, 1998; Drusano, 2004).

## 7 Lariocidin: a novel ribosome-targeting lasso peptide bridging Gram-positive and Gram-negative resistance

The rise of multidrug-resistant bacteria, commonly referred to as superbugs, has become one of the most serious global threats to modern medicine. The World Health Organization continues to monitor this escalating crisis through its Bacterial Priority Pathogens List, which now includes 24 antibiotic-resistant pathogens across 15 bacterial families, stratified into critical, high, and medium priority tiers These pathogens have developed highly effective defense mechanisms that allow them to withstand even the most potent antimicrobial agents, thereby rendering many established antibiotic therapies increasingly ineffective (O’Neill, 2016; World Health Organization, 2024).

Lasso peptides belong to the family of ribosomally synthesized and post translationally modified peptides and are defined by a characteristic lasso architecture in which a macrolactam ring encircles a threaded C terminal tail, creating a compact and interlocked topology. They share notable structural features with cyclotides, knottins and defensins, where disulfide bonds and, in the case of cyclotides, head to tail cyclization stabilize the peptide into a highly resilient conformation. Lasso peptides display a wide range of biological activities, including enzyme inhibition and antimicrobial, antiviral and anticancer effects. Their distinctive topology provides exceptional thermal and proteolytic stability, while their modular nature makes them particularly amenable to molecular engineering, offering advantages over conventional linear peptides (Al Musaimi, 2024; Mucha et al., 2025).

Lariocidin (LAR) is produced by *Paenibacillus* sp. M2 and characterized by a tightly knotted, sterically constrained structure. It functions as a broad-spectrum antibacterial compound that inhibits bacterial growth by binding to a unique site on the small ribosomal subunit. This interaction blocks ribosomal translocation and induces miscoding during protein synthesis. Lariocidin is notable for its resistance to common antimicrobial resistance mechanisms, low propensity for spontaneous resistance, lack of human cell toxicity, and potent *in vivo* activity against multidrug-resistant pathogens such as *Acinetobacter baumannii*. It represents the first known ribosome-targeting lasso peptide and a promising scaffold for developing new antibacterial therapeutics (Barrett and Mitchell, 2024; Wright et al., 2024).

Although structurally diverse, most ribosome-targeting antibiotics bind to a limited number of well-characterized regions, including the decoding center, the peptidyl transferase center, and the nascent peptide exit tunnel. The increasing prevalence of resistance mechanisms, particularly rRNA modifications and drug-inactivating enzymes, has compromised the effectiveness of agents acting on these conserved sites, often leading to cross-resistance across multiple antibiotic classes. These limitations highlight the urgent need to develop compounds that engage previously unexplored ribosomal regions through distinct structural and mechanistic approaches, thereby expanding therapeutic options against multidrug-resistant pathogens (Lin et al., 2018; Wright et al., 2024).

Lariocidin exhibits potent bactericidal activity; however, despite its high content of positively charged residues, its mechanism does not appear to involve membrane disruption. Although initially hypothesized to act similarly to cationic peptides such as colistin (Hancock and Rozek, 2002), experimental data showed that treatment reduces viable E. coli counts without causing cell lysis, membrane permeabilization, depolarization, or detectable morphological changes. These findings suggest that its antibacterial effect is mediated by an intracellular target. Supporting this conclusion, fluorescently labelled lariocidin retaining full antimicrobial activity was observed to accumulate within the bacterial cytoplasm, consistent with a non-membranolytic mechanism of action [145, 148 Moreover, it retains antibacterial efficacy in a murine model of *Acinetobacter baumannii* infection. Further pharmacological and translational evaluation will be essential to determine its clinical applicability (Jangra et al., 2025).

Pharmacokinetic and pharmacodynamic properties remain incompletely characterized. As a peptide-based compound, lariocidin may face challenges related to systemic stability, tissue penetration, proteolytic degradation, and oral bioavailability. Optimization of dosing strategies and exposure–response relationships will be critical to determine whether sufficient target engagement can be achieved *in vivo*, particularly in infections involving high bacterial burden or biofilm-associated growth (Mahlapuu et al., 2016; Jangra et al., 2025).

Second, although initial studies suggest a low spontaneous frequency of resistance, evolutionary pressure in clinical settings may still select for adaptive mutations. Potential resistance pathways could include ribosomal target modifications, altered peptide uptake mechanisms, efflux upregulation, or enzymatic inactivation. Third, manufacturing scalability and cost considerations may influence clinical feasibility. Lasso peptides require specialized biosynthetic or synthetic production platforms, and large-scale manufacturing efficiency remains to be demonstrated. In addition, toxicity, immunogenicity, and long-term safety profiles require comprehensive evaluation before clinical translation (Fosgerau and Hoffmann, 2015; Mahlapuu et al., 2016).

The inclusion of lariocidin in this review is directly motivated by its mechanistic relevance to the Gram-positive/Gram-negative resistance landscape described in preceding sections. Conventional ribosome-targeting antibiotics are increasingly compromised by organism-specific resistance mechanisms: in Gram-positive pathogens such as *Staphylococcus aureus* and *Streptococcus pneumoniae*, 23S rRNA methylation mediated by Erm enzymes and mutations in ribosomal proteins L3/L4 constitute the dominant resistance pathways, whereas in Gram-negative organisms such as *Acinetobacter baumannii* and Enterobacterales, enzymatic inactivation of aminoglycosides and plasmid-borne 16S rRNA methyltransferases further limit ribosome-targeted therapy (Munita and Arias, 2016; Foster, 2017; Gomez et al., 2017). Lariocidin addresses both contexts simultaneously: by engaging a previously unexploited site on the small ribosomal subunit, it circumvents the resistance mechanisms that compromise existing agents in both Gram-positive and Gram-negative pathogens, and has demonstrated *in vivo* efficacy specifically against *Acinetobacter baumannii*, one of the most treatment-refractory Gram-negative ESKAPE pathogens, while retaining activity against Gram-positive organisms (Lin et al., 2018). Its introduction therefore represents a structurally novel approach to overcoming resistance mechanisms that are mechanistically distinct but clinically convergent across both bacterial groups (Munita and Arias, 2016; Lin et al., 2018).

While lariocidin and related ribosome-targeting lasso peptides represent a promising structural class, their clinical relevance currently remains preclinical and should be interpreted with caution. The available evidence, derived primarily from *in vitro* and murine infection models, has not yet been replicated across independent laboratories or extended to human pharmacokinetic studies, and conclusions regarding the low propensity for spontaneous resistance may not generalize to prolonged clinical exposure (Wright et al., 2024; Jangra et al., 2025). At present, no lasso peptide antibiotic has advanced to clinical trials, in contrast to next-generation β-lactamase inhibitors, which already address a larger share of the global carbapenemase burden (Ehmann et al., 2012; Hecker et al., 2015; Jangra et al., 2025). The principal knowledge gaps include the absence of human safety and tolerability data, uncertainty regarding manufacturing scalability at clinical scale, and the lack of comparative studies positioning lasso peptides relative to other antimicrobial peptide classes in terms of therapeutic index and resistance durability (Fosgerau and Hoffmann, 2015; Mahlapuu et al., 2016; Jangra et al., 2025).

## 8 Integrative perspective: linking resistance mechanisms, diagnostics, and emerging therapeutics across Gram-positive and Gram-negative bacteria

The resistance mechanisms described throughout this review do not operate in isolation, but rather form an interconnected network whose clinical impact is shaped by the fundamental biological differences between Gram-positive and Gram-negative bacteria (Munita and Arias, 2016; De Oliveira et al., 2020). In Gram-negative ESKAPE pathogens, the outer membrane provides a structural scaffold upon which enzymatic inactivation, porin loss, and RND efflux pump overexpression act synergistically, creating a multilayered resistance phenotype that is particularly difficult to overcome with existing agents (Pang et al., 2019; Shi et al., 2024; Mourabiti et al., 2025). In Gram-positive pathogens, the absence of an outer membrane shifts the resistance burden toward target-site alterations and horizontal gene transfer of resistance determinants such as *mecA* and *van* gene clusters, which spread rapidly within healthcare settings (Miller et al., 2014; Foster, 2017). Biofilm formation amplifies both resistance profiles: in Gram-negative organisms by adding matrix-limited drug diffusion atop existing permeability barriers, and in Gram-positive organisms by creating metabolically dormant persister populations that evade both antibiotics and host immune defenses (Franklin et al., 2011; Otto, 2013; Koo et al., 2017).

These mechanistic distinctions have direct and practical consequences for diagnosis and treatment. Phenotypic and molecular diagnostic strategies must be organism-group-aware: carbapenemase gene panels for Gram-negative pathogens, *mecA*/*vanA* screening for Gram-positive organisms, and pharmacodynamic optimization must account for how resistance mechanisms alter drug exposure at the target site differently in each group (Craig, 1998; Munita and Arias, 2016; van Belkum et al., 2020; Ren et al., 2022). The mutant selection window concept, for instance, has different implications in *Pseudomonas aeruginosa* (where efflux upregulation can shift MIC distributions rapidly under subtherapeutic exposure) compared to MRSA (where target-site mutations accumulate more slowly but are difficult to reverse once established) (Zhao and Drlica, 2001; Drusano, 2004; Pang et al., 2019).

Lariocidin emerges from this landscape as a mechanistically rational response: by targeting a ribosomal site that has not been subject to the evolutionary pressure of existing antibiotics, it bypasses the organism-specific resistance pathways that compromise conventional ribosome-targeting agents in both bacterial groups (Lin et al., 2018; Jangra et al., 2025). Its broad-spectrum activity across Gram-positive and Gram-negative multidrug-resistant pathogens positions it as a potential bridging agent across the resistance divide that currently fragments our therapeutic armamentarium (Mahlapuu et al., 2016; Jangra et al., 2025). Realizing its clinical potential will require integrating the pharmacodynamic, diagnostic, and resistance-surveillance frameworks discussed throughout this review, underscoring that progress against AMR demands not isolated innovations, but a coherent, mechanistically informed strategy that spans both bacterial groups (Craig, 1998; Drusano, 2004; Munita and Arias, 2016; Lin et al., 2018).

## 9 Conclusions

Antimicrobial resistance (AMR) among clinically significant Gram-positive and Gram-negative pathogens continues to pose a critical therapeutic challenge, driven by mechanistically distinct yet epidemiologically convergent resistance pathways and the widespread misuse of antibiotics. In Gram-positive organisms, such as *Staphylococcus aureus* and Enterococcus spp., resistance is predominantly mediated by target-site alterations, including PBP2a-mediated β-lactam resistance in MRSA and van-gene-cluster-mediated glycopeptide resistance in VRE. In Gram-negative ESKAPE pathogens such as Klebsiella pneumoniae, Acinetobacter baumannii, and Pseudomonas aeruginosa, resistance arises from a more complex interplay of carbapenemase production, efflux pump overexpression, porin loss, and biofilm-associated tolerance, often acting synergistically. These differences have direct implications for diagnostics and therapy: phenotypic and molecular detection strategies must be tailored to organism-specific resistance determinants, and pharmacodynamic optimization requires distinct approaches for Gram-positive versus Gram-negative infections. Biofilm-associated resistance, while broadly conserved, differs in matrix composition and antibiotic tolerance mechanisms between the two groups, further complicating eradication strategies.

The growing resistance among ESKAPE pathogens highlights the urgent need for robust antimicrobial stewardship, rapid diagnostic methods, novel therapeutic agents, and coordinated public health interventions to prevent the erosion of current treatment efficacy.

Lariocidin represents a significant addition to the growing class of ribosomally-synthesized and post- translationally modified peptides with clinically relevant antimicrobial activity. Its distinctive lasso topology, combined with a unique ability to bind an unexploited site on the small ribosomal subunit, positions it as a promising candidate for the development of next-generation antibiotics. Unlike many conventional agents, lariocidin is not compromised by common resistance mechanisms, shows a low likelihood for the emergence of spontaneous resistance and demonstrates potent activity against a broad range of bacterial pathogens. Its lack of toxicity toward human cells and its efficacy *in vivo* further strengthen its therapeutic potential. Altogether, lariocidin provides a novel chemical scaffold for antibacterial drug design and highlights the value of structurally unconventional peptide frameworks in addressing the urgent global challenge of antimicrobial resistance.
